# Hydropathicity-based prediction of pain-causing NaV1.7 variants

**DOI:** 10.1186/s12859-021-04119-2

**Published:** 2021-04-23

**Authors:** Makros N. Xenakis, Dimos Kapetis, Yang Yang, Monique M. Gerrits, Jordi Heijman, Stephen G. Waxman, Giuseppe Lauria, Catharina G. Faber, Ronald L. Westra, Patrick J. Lindsey, Hubert J. Smeets

**Affiliations:** 1 Department of Toxicogenomics, Section Clinical Genomics, Maastricht University, PO Box 616, 6200 MD Maastricht, The Netherlands; 2 Research School for Mental Health and Neuroscience (MHeNS), Maastricht University, PO Box 616, 6200 MD Maastricht, The Netherlands; 3 Neuroalgology Unit, Fondazione IRCCS Istituto Neurologico “Carlo Besta”, Via Celoria 11, 20133 Milan, Italy; 4 Department of Medicinal Chemistry and Molecular Pharmacology, Purdue University College of Pharmacy, West Lafayette, IN 47907 USA; 5 Purdue Institute for Integrative Neuroscience, West Lafayette, IN 47907 USA; 6 Department of Clinical Genetics, Maastricht University Medical Center, PO box 5800, 6202 AZ Maastricht, The Netherlands; 7 Department of Cardiology, CARIM School for Cardiovascular Diseases, Maastricht University, PO Box 616, 6200 MD Maastricht, The Netherlands; 8 Department of Neurology and Center for Neuroscience and Regeneration Research, Yale University School of Medicine, New Haven, CT 06510 USA; 9 Rehabilitation Research Center, Veterans Affairs Connecticut Healthcare System, West Haven, CT 06516 USA; 10 Department of Biomedical and Clinical Sciences “Luigi Sacco”, University of Milan, Via G.B. Grassi 74, 20157 Milan, Italy; 11 Department of Neurology, Maastricht University Medical Center, PO Box 5800, 6202 AZ Maastricht, The Netherlands; 12 Department of Data Science and Knowledge Engineering, Maastricht University, PO Box 616, 6200 MD Maastricht, The Netherlands; 13 Research School for Oncology and Developmental Biology (GROW), Maastricht University, PO Box 616, 6200 MD Maastricht, The Netherlands

**Keywords:** NaV1.7, Missense mutations, Pain, Atomic hydropathicity, Computational modeling, Cumulative hydropathic topology, Scaling, Pathogenicity prediction

## Abstract

**Background:**

Mutation-induced variations in the functional architecture of the NaV1.7 channel protein are causally related to a broad spectrum of human pain disorders. Predicting in silico the phenotype of NaV1.7 variant is of major clinical importance; it can aid in reducing costs of in vitro pathophysiological characterization of NaV1.7 variants, as well as, in the design of drug agents for counteracting pain-disease symptoms.

**Results:**

In this work, we utilize spatial complexity of hydropathic effects toward predicting which NaV1.7 variants cause pain (and which are neutral) based on the location of corresponding mutation sites within the NaV1.7 structure. For that, we analyze topological and scaling hydropathic characteristics of the atomic environment around NaV1.7’s pore and probe their spatial correlation with mutation sites. We show that pain-related mutation sites occupy structural locations in proximity to a hydrophobic patch lining the pore while clustering at a critical hydropathic-interactions distance from the selectivity filter (SF). Taken together, these observations can differentiate pain-related NaV1.7 variants from neutral ones, i.e., NaV1.7 variants not causing pain disease, with 80.5$\%$ sensitivity and 93.7$\%$ specificity [area under the receiver operating characteristics curve = 0.872].

**Conclusions:**

Our findings suggest that maintaining hydrophobic NaV1.7 interior intact, as well as, a finely-tuned (dictated by hydropathic interactions) distance from the SF might be necessary molecular conditions for physiological NaV1.7 functioning. The main advantage for using the presented predictive scheme is its negligible computational cost, as well as, hydropathicity-based biophysical rationalization.

**Supplementary Information:**

The online version contains supplementary material available at 10.1186/s12859-021-04119-2.

## Introduction

Voltage-gated sodium channels (NaVChs) are pore-forming proteins embedded in cell membranes. They are members of the ion channels superfamily and their main physiological role is to control transport of sodium ions into the cell. The human NaV1.7 channel is encoded by the *SCN9A* gene and is preferentially expressed in peripheral neurons (e.g., dorsal root ganglion (DRG) nociceptors) responsible for networking pain signals. The structure of the NaV1.7 $\alpha$-subunits is that of a pore-forming tetramer via assembly of four heterogeneous domains (DI-DIV). Three intracellular linkers (L1-L3) form structural interconnections among subsequent domains. Each domain comprises six transmembrane helices (S1-S6) organized into a pore module (PM) forming an ion-conduction pathway coupled with a voltage-senor (VS). Mechanistic description of NaV1.7’s function is that VSs react to extracellular changes in ionic concentrations by moving outwards thus exerting a pulling force upon the PM which opens the channel pore. Closed-to-open conformational change leads to channel activation, i.e., renders it conductive to sodium ions. Missense mutations in the *SCN9A* gene can destabilize the NaV1.7’s functional architecture thus disrupting physiological sodium-ions current and, consequently, deregulate flow of sodium ions through the pore. At a cellular level, these genetically-caused destabilizations can affect neuronal excitability by inducing a gain-of-function (GOF) effect, i.e., by increasing the net sodium-ions currents, thus triggering a wide spectrum of pain diseases such as inherited erythromelalgia (IEM) [1–30], paroxysmal extreme pain disorder (PEPD) [31–37] and small fiber neuropathy (SFN) [38–42]. A proof of concept for the GOF-pain correlation hypothesis came from identification of missense *SCN9A*-gene mutations inducing a loss-of-function (LOF) effect, i.e., decreasing net sodium-ions current, that is causally related to clinical symptoms of loss of pain sensation [43–45].

Hydropathic interactions (HIs) represent a summary of fundamental molecular interactions [46] driving molecular phenomena such as protein folding, protein hydrophobic-core stability, self-assembly of amphiphilic molecules, and “dewetting” of molecular surfaces (for a review in HIs-driven phenomena see [47]). Within the field of ion channels research, experimental and computational studies have shown that pore-lining patches of hydrophobic residues are crucial regulators of, both, pore’s gating behavior (a phenomenon termed as “hydrophobic gating” [48]) and channel stability [49], e.g., via formation of hydrogen-bonds networks expanding through their surroundings [50, 51]. Crucially, hydrophobic patches (HPs) are widely conserved across voltage-gated channels and often associated with the formation of centrally-located cavity (CC) [52]. Mutations perturbing this hydrophobic motif can lead to drastic changes of net ion currents [53–56].

Computational modeling of HIs combined with biophysical observations extracted from in vitro NaVCh pathophysiological characterization can propel our understanding of mechanistic linkages between mutation-induced perturbations and human pain pathophysiology. Key-studies toward this direction were these of Lampert et al [57], and of Yang et al [58] demonstrating how the F1449V mutation, and the in-frame-deletion L955Del, respectively, can disrupt a hydrophobic ring stabilizing the putative activation gate (AG) of the NaV1.7 thus acting as disease-causing molecular triggers. Moreover, computational modeling successfully deduced an energetic coupling between two different IEM-related mutations foreseen by their geometrical proximity in NaV1.7 structure [7]. A question that naturally arose from these studies was whether a detailed examination of HIs network characteristics within a NaV1.7 structure can reveal statistically-significant but also biophysically-relevant differentiations among the WT structure and its variants. This question was probed by Kapetis et al [59]; a network-theoretical computational framework was introduced in order to capture changes in interatomic HIs within a NaV1.7 structural model induced by pain-related mutations. The study reported on a betweenness centrality network measure achieving a statistically-important differentiation of pain-related variants from a collection of neutrals, i.e., variants not causing pain disease. Notably, this approach highlighted the prominent role that HIs play in NaV1.7’s stability and reported on plausible mutation-mechanism scenarios disrupting hydrophobic contacts among neighboring and distant residues. Another remark on the multi-scale nature of HIs was made by the authors of [60] suggesting that a pathogenic mutation in the *KCNA*1 gene encoding the human voltage-gated potassium channel KV1.1 can de-tune HIs equilibrium (and, consequently, destabilize KV1.1’s pre-open conformation) implying that mutation-induced perturbation effects can destroy finely-tuned network-like HIs expanding throughout the structure as a whole. Interestingly, the fine-tuning hypothesis was proposed also for the NaV1.7; a recent study employing a machine learning (MLE) computational pipeline for predicting NaV1.7’s variant pathogenicity suggested that the fine-tuning of the NaV1.7 channel is so delicate that limits classification accuracy of practically any computational approach [61]. Taken together, these observations highlight the highly-cooperative nature of HIs [46] and suggest that even small changes in the hydropathic spatial distribution profile of a channel structure can have a detrimental impact on the functional architecture which, in turn, might induce clinically-observed alterations of electrophysiology.

Following [59, 61], this study aims at probing the finely-tuned hypothesis for the NaV1.7’s atomic hydropathic environment towards predicting whether a NaV1.7 variant causes pain or not. We demonstrate our approach on a closed-state NaV1.7 structural model (first presented in [7] and later also used in [30, 58]) by investigating cumulative, i.e., scale-dependent, hydropathic properties of its porous atomic environment in relation to structural locations of missense *SCN9A*-gene mutations. In order to tackle spatial complexities emerging from the highly-cooperative nature of HIs we adopt a modeling approach rooted in the hypothesis that proteins can be represented as self-organized criticality (SOC) [62] archetypes; protein structures are thought to have been evolutionary optimized with respect to extrema in some thermodynamic property (or properties) capturing a qualitative reorganization of the atomic environment [63, 64]. The intra-channel locations where these macroscopic thermodynamic changes take place correspond to so-called critical points of the atomic structure [63, 64]. The highly-cooperative nature of HIs has placed structure-retrieved hydropathic properties in the epicenter of SOC hypothesis [63–66]). It is important to note that computational evidence for a universal hydrophobic-to-hydrophilic (or inside-outside with “inside” referring to the hydrophobic core, and “outside” referring to the hydrophilic exterior) spatial transition in protein systems was first provided before the formulation of the SOC hypothesis (see [67]). Departing from this phenomenological basis, we here utilize the finite-size scaling analysis methodologies presented in [68, 69] for screening hydropathic morphology around NaV1.7’s pore [68] toward identification of critical points associated with NaV1.7’s functional architecture. Biophysical relevance of retrieved observations is justified not only in terms of the scale-invariance of a carefully-chosen cumulative hydropathicity-property function but also with respect to conserved structural NaV features such as the PM-VSs spatial transition [70, 71] and the architecture of the selectivity filter (SF) [72]. In particular, we demonstrate that the atomic cumulative distribution function around NaV1.7’s pore exhibits a sigmoid profile with inflection points matching closely the conserved PM-VSs spatial transition. This provides a rigorous description of atom-packing geometry and, consequently, a macroscopic partitioning of the atomic environment around the pore allowing for mapping the spatial profile of the atomic cumulative hydropathicity-property function and mutation sites on two dimensions. The SOC hypothesis is then accepted (or rejected) depending on whether the cumulative hydropathicity-property function is globally maximized and exhibits power-law-like scaling behavior in the vicinity of the inflection point (or not).

Our mapping procedures reveal the formation of a HP incorporating NaV1.7’s CC and AG. We report on two “hot” map areas attracting pain-related mutation sites distributed along HP’s boundary. Probing the SOC hypothesis for HIs around NaV1.7’s pore reveals that “hot” structural locations tend to cluster at a distance of $\sim$33.4 Å from the SF. Stability implications of these observations can be deduced by considering that in the vicinity of the critical point the range and intensity of HIs increase in a power-law fashion thus favoring amplification and propagation of mutation-induced perturbations peripherally to the HP and at critical HIs-distance from the SF thus not directly affecting neither of them. The clinical translational value of our findings is tested by predicting pathogenicity of 84 NaV1.7 variants; a weighted average of HP- and SF-related distance metrics can classify up to 29 (out of 36) pain-related variants and 45 (out of 48) neutral variants correctly.

## Methods

All computations were performed in R [73] environment unless stated differently.

### 3D structure preparation

The NaV1.7 atomic structural model used throughout this study was constructed via homology modeling procedures based on the pre-open NaVAb template [PDB code: 3RVY] [74] according to [7]. In short, the first step undertaken was to generate structural models of the four transmembrane domains (DI-DIV) by utilizing the membrane-bound protein predication algorithm GPCR-ITASSER [75–77]. Then, each domain was aligned upon the pre-open NaVAb template by using the TM-align algorithm [78]. Finally, the four transmembrane domains were placed together in a clockwise order viewed from extracellular side (ES) according to [79, 80], and the resulting four domain complex structural model was refined by fragment-guided molecular dynamics simulations aiming at removing interdomain clashes, optimizing hydrogen atoms network, and, consequently, increasing model quality [75, 76, 81]. The retrieved model consists of 1140 protonated amino acids (DI: P229:K417, DII: P839:T972, DIII: M1296:G1461, DIV: K1617:T1763). A comparison via superposition of the retrieved model with a recently resolved cryo-electron microscopy (cryo-EM) NaV1.7 structure [PDB code: 6J8J] [82] is presented in Additional file 1: S1.

In continuation, principal axes of the NaV1.7 model structure were estimated by using the VMD software [83]. A global coordinate system $(\hat {\mathbf{x}},\hat {\mathbf{y}},\hat {\mathbf{z}})$ was introduced with its center at *O* and the NaV1.7’s principal pore axis, i.e., the axis approximating the direction of the channel’s pore, was aligned with the *z*-axis with orientation from the ES toward the intracellular side (IS) with respect to $\hat{\mathbf {z}}$. The atomic center $\varvec{\mathrm{e}}=\frac{1}{M}\sum _{i=1}^{N_{c}}m_{i}\cdot \varvec{\mathrm{c}}_{i}$ of the 3D structure was set to overlap with *O*, where $\varvec{\mathrm{c}}_{i}=(\mathrm{c}_{x,i},\mathrm{c}_{y,i},\mathrm{c}_{z,i})$ is the atomic center of the *i*th atom, $m_{i}$ is the mass of the *i*th atom, $N_{c}=18567$ is the total number of atoms, and $M=\sum _{i=1}^{N_{c}}m_{i}$ is the total molecular mass (values of atomic masses are the same as [68, 69]).

### Geometrical characteristics of the pore

We navigated through the skewed NaV1.7’s pore by introducing a set of pore points ${\mathbf{p}}\in P$ (see Additional file 1: S2). The pore radius at ${\mathbf{p}}$ is given by [? ]

m2 $$\begin{aligned} R({\mathbf{p}}) = \underset{i=1,2,\ldots,N_{c}}{min}\{||\varvec{\mathrm{c}}_{i} -{\mathbf{p}}|| - vdW_{i}\} \end{aligned}$$

where $||\cdot ||$ is the euclidean norm and vdW$_{i}$ is the van der Waals radius of the *i*th atom (values of van der Waals radii are the same as [68, 69]). The distance between ${\mathbf{p}}$ and its nearest neighbor atom corresponds then to

m3 $$\begin{aligned} D({\mathbf{p}}) = \underset{i=1,2,\ldots,N_{c}}{min}\{||\varvec{\mathrm{c}}_{i} -{\mathbf{p}}||\} \end{aligned}$$

and the outer surface radius at ${\mathbf{p}}$ is given by [68, 69]

m4 $$\begin{aligned} L({\mathbf{p}}) = \underset{i=1,2,\ldots,N_{c}}{max}\{||\varvec{\mathrm{c}}_{i} -{\mathbf{p}}|| + vdW_{i}\} \end{aligned}$$

where the unit of measurement for $R({\mathbf{p}})$, $D({\mathbf{p}})$ and $L({\mathbf{p}})$ is expressed in [Å].

### Finite-size sampling around the pore

The atomic environment around ${\mathbf{p}}$ is sampled with concentric spheres placed at ${\mathbf{p}}$ of increasing radius [68, 69]

m5 $$\begin{aligned} l_{\alpha }({\mathbf{p}}) = D({\mathbf{p}}) +\alpha \cdot \frac{L({\mathbf{p}})-D({\mathbf{p}})}{K_{\alpha }}\,\,\,\rm{for}\,\,\,\alpha =1,2,\ldots,K_{\alpha }\rightarrow \infty \end{aligned}$$

where $K_{\alpha }$ is the total number of sampling spheres and $\alpha$ denotes the index of the sampling sphere (i.e., the scaling index). $l_{\alpha }({\mathbf{p}})$ indicates the size, i.e., molecular scale, of the spherical cluster of atoms around ${\mathbf{p}}$ in [Å] and $L({\mathbf{p}})$ the finite channel size measured with respect to ${\mathbf{p}}$. Accordingly, the atomic cumulative distribution function (CDF) at ${\mathbf{p}}$ is given by [68, 69]

m6 $$\begin{aligned} N({\mathbf{p}},l_{\alpha }({\mathbf{p}})) = \sum _{i=1}^{N_{c}} \theta (l_{\alpha }({\mathbf{p}}) - ||\varvec{\mathrm{c}}_{i} -{\mathbf{p}}||) \end{aligned}$$

where $\theta (\cdot )$ is the heaviside function. Note that $N({\mathbf{p}},l_{\alpha }({\mathbf{p}}))$ essentially describes how atoms are packed around ${\mathbf{p}}$. In computational practice $K_{\alpha }$ is set to be “large enough” approximating the continuous case via dense sampling.

### Mathematical modeling of atomic CDF

Modeling of the atomic CDF was performed by employing the GROFIT routine [84]. Hypothesis underlying this modeling approach is that the atomic CDF for a given ${\mathbf{p}}$ follows a sigmoid pattern. Hypothesis is approved if GROFIT manages to fit any of available candidate models to the CDF data. Available candidate models are a re-parametrized algebraic forms [85] of the Logistic model [86]

m7 $$\begin{aligned} n_{LOG}({\mathbf{p}},l_{\alpha }({\mathbf{p}})) = A({\mathbf{p}}) \cdot \big \{ 1 + \text {exp}\big ( \frac{4\cdot t({\mathbf{p}})}{A({\mathbf{p}})} \cdot \big (s({\mathbf{p}}) - l_{\alpha }({\mathbf{p}})) + 2 \big ) \big \}^{-1} \end{aligned}$$,

of the Gompertz model [87]

m8 $$\begin{aligned} n_{GOM}({\mathbf{p}},l_{\alpha }({\mathbf{p}})) = A({\mathbf{p}}) \cdot \text {exp} \big ( -\text {exp} (\frac{e\cdot t({\mathbf{p}})}{A({\mathbf{p}})} \cdot (s({\mathbf{p}}) - l_{\alpha }({\mathbf{p}})) + 1) \big ) \end{aligned}$$

with $e=\mathrm{exp}(1)$, of the the modified Gompertz model [85]

m9 $$\begin{aligned} n_{MGOM}({\mathbf{p}},l_{\alpha }({\mathbf{p}})) &=A({\mathbf{p}})\cdot \text {exp} \big ( -\text {exp} \big ( \frac{e\cdot t({\mathbf{p}})}{A({\mathbf{p}})} \cdot (s({\mathbf{p}}) - l_{\alpha }({\mathbf{p}})) + 1 \big ) \big )\\&+ A({\mathbf{p}}) \cdot \text {exp} \big ( w({\mathbf{p}}) \cdot (l_{\alpha }({\mathbf{p}}) - l_{shift}({\mathbf{p}})) \big ) \end{aligned}$$

and of the Richards model [88]

m10 $$\begin{aligned} n_{{RIC}} ({\mathbf{p}},l_{\alpha } ({\mathbf{p}})) & = A({\mathbf{p}}) \cdot \{ 1 + \tilde{q}({\mathbf{p}}) \cdot b({\mathbf{p}}) \cdot {\text{exp}}( - k({\mathbf{p}}) \cdot l_{\alpha } ({\mathbf{p}}))\} ^{{ - 1/\tilde{q}({\mathbf{p}})}} \\ & \quad {\text{with }}b({\mathbf{p}}) = {\text{exp}}(1 + \tilde{q}({\mathbf{p}}) + k({\mathbf{p}}) \cdot s({\mathbf{p}})){\text{ and }}k({\mathbf{p}}) = \frac{{t({\mathbf{p}})}}{{A({\mathbf{p}})}} \cdot (1 + \tilde{q}({\mathbf{p}}))^{{1 + 1/\tilde{q}({\mathbf{p}})}} \\ \end{aligned}$$

were fitted on $N({\mathbf{p}},l_{\alpha }({\mathbf{p}}))$ along $l_{\alpha }({\mathbf{p}})$-direction where $\{A({\mathbf{p}}),t({\mathbf{p}}),s({\mathbf{p}}),\tilde{q}({\mathbf{p}}),w({\mathbf{p}}),l_{shift}({\mathbf{p}})\}$ are model parameters. The candidate model that best fits the atomic CDF is then selected based on minimization of an Akaike information criterion (see [84] for algorithmic details).

Following [69], model parameters interpretation was performed with respect to the inflection point

m11 $$\begin{aligned} \xi ({\mathbf{p}}) = \{ l_{\alpha }({\mathbf{p}})\,\,\big | \,\,\frac{\partial ^{2} n({\mathbf{p}},l_{\alpha }({\mathbf{p}}))}{\partial l_{\alpha }({\mathbf{p}})^{2}} = 0 \} \end{aligned}$$

that determines the location along $l_{\alpha }({\mathbf{p}})$-direction where the radial distribution function (RDF), $\frac{\partial n({\mathbf{p}},l_{\alpha }({\mathbf{p}}))}{\partial l_{\alpha }({\mathbf{p}})}$, maximizes. The RDF maximum value is given by the parameter $t({\mathbf{p}})$ accounting for the maximum atom-packing rate (or, equivalently, for the maximum atomic density) around ${\mathbf{p}}$. Parameter $A({\mathbf{p}})$ is the asymptote value of the fitted model, i.e., $n({\mathbf{p}},l_{\alpha }({\mathbf{p}})\rightarrow \infty )=A({\mathbf{p}})$, describing what happens when $L({\mathbf{p}})$ becomes arbitrary large. Parameter $s({\mathbf{p}})$ determines the location along $l_{\alpha }({\mathbf{p}})$-direction where the lag atom-packing domain ends, i.e., its size. Notably, parameter $t({\mathbf{p}})$ can be expressed in terms of the ratio $t({\mathbf{p}})=\frac{A({\mathbf{p}})}{os({\mathbf{p}}):=o({\mathbf{p}})-s({\mathbf{p}})}$ with $o({\mathbf{p}})$ determining the location along $l_{\alpha }({\mathbf{p}})$-direction where the asymptote atom-packing domain begins. Parameter $\tilde{q}({\mathbf{p}})$ affects the shape of the Richards model curve, as well as, the location of the inflection point thus plays the role of the summary atom-packing parameter. Parameters $w({\mathbf{p}})$, and $l_{shift}({\mathbf{p}})$ of the modified Gompertz model indicate the location, and the slope, respectively, of a second increase in the modified Gompertz model curve [84]. The Logistic and the Gompertz model are retrieved from the Richards model for $\tilde{q}({\mathbf{p}})=1$, and for $\tilde{q}({\mathbf{p}})\rightarrow 0$, respectively, as shown in [89], thus they are considered as special cases of the Richards model.

### Cumulative hydropathicity-property functions

The hydropathic density of the atomic environment around ${\mathbf{p}}$ was approximated in terms of [68]

m12 $$\begin{aligned}&m^{(0)}({\mathbf{p}}, l_{\alpha }({\mathbf{p}})) = \frac{h^{(0)}({\mathbf{p}}, l_{\alpha }({\mathbf{p}}))}{N({\mathbf{p}},l_{\alpha }({\mathbf{p}}))}\sim kcal/(mol\equiv atom) \\&\text { with }h^{(0)}({\mathbf{p}}, l_{\alpha }({\mathbf{p}}))=\sum _{i=1}^{N_{c}} \theta (l_{\alpha }({\mathbf{p}}) - ||\varvec{\mathrm{c}}_{i} -{\mathbf{p}}||)\cdot HI^{w}_{i} \end{aligned}$$

where $\varvec{h}^{(0)}({\mathbf{p}}, l_{\alpha }({\mathbf{p}}))$ corresponds to the cumulative zero-order hydropathic pore moment function [90] with $HI^{w}_{i}=HI_{i} + w_{i}$ representing the *i*th atomic hydrophobic index in accordance with the Kapcha&Rossky atomic hydropathic scale [91] with additive Gaussian noise $w_{i}\in \mathcal {N}(\mu =0,\sigma =0.001)$. The superscript “(0)” indicates the moment order.

The hydropathic interatomic interaction strength (HIIS) at ${\mathbf{p}}$, i.e., the average interaction strength between an atomic component found within the cluster of size $N({\mathbf{p}}, l_{\alpha }({\mathbf{p}}))$ and its surroundings, was approximated in terms of the hydropathic imbalance (or interaction strength) pore function [68, 69]

m13 $$\begin{aligned}&{\vec{\varvec{m}}}^{(1)}({\mathbf{p}}, l_{\alpha }({\mathbf{p}})) = \frac{{\vec{\varvec{h}}}^{(1)}({\mathbf{p}}, l_{\alpha }({\mathbf{p}}))}{N({\mathbf{p}},l_{\alpha }({\mathbf{p}}))}\sim {\mathrm{kcal}}\!\cdot\!\AA / ({\mathrm{mol}}\equiv {\mathrm{atom}}) \\&{\text { with }}{{\vec{\varvec{h}}}}^{(1)}({\mathbf{p}}, l_{\alpha }({\mathbf{p}}))=\sum _{i=1}^{N_{c}}\theta (l_{\alpha }({\mathbf{p}}) - ||{\mathbf{c}}_{i} -{\mathbf{p}}||)\cdot HI^{w}_{i}\cdot {{\vec{\varvec{r}}}} _{{\mathrm{p}},i} \\&= \underbrace{h^{(1)}_{x}({\mathbf{p}},l_{\alpha }({\mathbf{p}}))\cdot  {\hat{\mathbf{x}}}+ h^{(1)}_{y}({\mathbf{p}},l_{\alpha }({\mathbf{p}}))\cdot  {\hat{\mathbf{y}}}}_{{\vec{\varvec{h}}}^{(1)}_{xy}({\mathbf{p}},l_{\alpha }({\mathbf{p}}))}+ \underbrace{h^{(1)}_{z}({\mathbf{p}},l_{\alpha }({\mathbf{p}}))\cdot {\hat{\mathbf{z}}}} _{{\vec{\varvec{h}}}^{(1)}_{z}({\mathbf{p}},l_{\alpha }({\mathbf{p}}))} \end{aligned}$$

where ${\vec{\varvec{h}}}^{(1)}({\mathbf{p}}, l_{\alpha }({\mathbf{p}}))$ corresponds to the cumulative first-order hydropathic pore moment function [90] quantifying the hydropathic inter-cluster interaction strength (HIcIS) with ${\vec{\varvec{r}}}_{\mathrm{p},i}$ being the vector from ${\mathbf{p}}$ to $\varvec{\mathrm{c}}_{i}$. The superscript “(1)” indicates the moment order.

Introduction of a weak noise source $w_{i}$ practically guarantees that scalars $|h^{(0)}({\mathbf{p}},l_{\alpha }({\mathbf{p}}))|$ and $||{\vec{\varvec{h}}}^{(1)}({\mathbf{p}},l_{\alpha }({\mathbf{p}}))||$ are non-zero for every combination of ${\mathbf{p}}$ and $l_{\alpha }({\mathbf{p}})$ while their scaling behavior remains practically unaffected. Throughout this study we consider pore’s physichochemical field characteristics to be adequately described in terms of the HIIS axial field component, ${\vec{\varvec{m}}}^{(1)}_{z}({\mathbf{p}},l_{\alpha }({\mathbf{p}}))={\vec{\varvec{h}}}^{(1)}_{z}({\mathbf{p}},l_{\alpha }({\mathbf{p}}))/N({\mathbf{p}}, l_{\alpha }({\mathbf{p}}))=m^{(1)}_{z}({\mathbf{p}},l_{\alpha }({\mathbf{p}}))\cdot {\hat{\mathbf{z}}}$ given that the magnitude of the radial field component, $||{\vec{\varvec{h}}}^{(1)}_{xy}({\mathbf{p}},l_{\alpha }({\mathbf{p}}))||$, is statistically expected to remain always smaller than $||{\vec{\varvec{m}}}^{(1)}_{z}({\mathbf{p}},l_{\alpha }({\mathbf{p}}))||$ after a cut-off, lag-domain scale and, thereafter, to gradually shrink (see Additional file 1: S3). Accordingly, we focus only on the scaling behavior and topology of the HIIS axial field component which can occupy only two orientation-states; an “in” orientation-state which is characterized by ${\vec{\varvec{m}}}^{(1)}_{z}({\mathbf{p}},l_{\alpha }({\mathbf{p}}))$ pointing towards the intracellular side (IS), i.e., by $m^{(1)}_{z}({\mathbf{p}},l_{\alpha }({\mathbf{p}}))>0$, and an “out” orientation-state which is characterized by ${\vec{\varvec{m}}}^{(1)}_{z}({\mathbf{p}},l_{\alpha }({\mathbf{p}}))$ pointing towards the ES, i.e., by $m^{(1)}_{z}({\mathbf{p}},l_{\alpha }({\mathbf{p}}))<0$. Topological changes in HIIS axial field component are detected according to the algorithmic scheme presented in [68] (see Additional file 1: S4).

### Finite-size scaling of HIIS axial field component

In accordance to [69], a scale-invariant interval of the HIIS axial field component corresponds to combinations of ${\mathbf{p}}$ with $\alpha$ for which the power-law approximation

m14 $$\begin{aligned} ||{\vec{\varvec{m}}}^{(1)}_{z}({\mathbf{p}},l_{\alpha }({\mathbf{p}}))|| \sim l_{\alpha }({\mathbf{p}})^{\gamma ({\mathbf{p}})} \end{aligned}$$

is accurately satisfied indicating that HIIS axial field component stabilizing the cluster of $N({\mathbf{p}},l_{\alpha }({\mathbf{p}}))$ atoms around ${\mathbf{p}}$ span a range up to $\sim l_{\alpha }({\mathbf{p}})$ Å. Sign of $\gamma ({\mathbf{p}})$ quantifies the rate at which intensity and range of HIIS axial field component increase or decrease for increasing atomic cluster size. From a HIs-network standpoint, $\gamma ({\mathbf{p}})$ indicates whether HIs network interconnectivity is up- or down-regulated, i.e., whether HIs contacts, e.g., hydrogen bonds, are created or destroyed within the structure. The energy levels associated with HIs contacts creation (or destruction) guaranteeing stability of the atomic cluster can then be approximated by

m15 $$\begin{aligned} U({\mathbf{p}},l_{\alpha }({\mathbf{p}})){:}=||{\vec{\varvec{h}}}^{(1)}_{z}({\mathbf{p}},l_{\alpha }({\mathbf{p}}))||/l_{\alpha }({\mathbf{p}}) \sim N({\mathbf{p}},l_{\alpha }({\mathbf{p}}))\cdot l_{\alpha }({\mathbf{p}})^{\gamma ({\mathbf{p}})-1} \end{aligned}$$

measured in kcal/(mol$\equiv$atomic cluster).

## Results

**Fig. 1:**
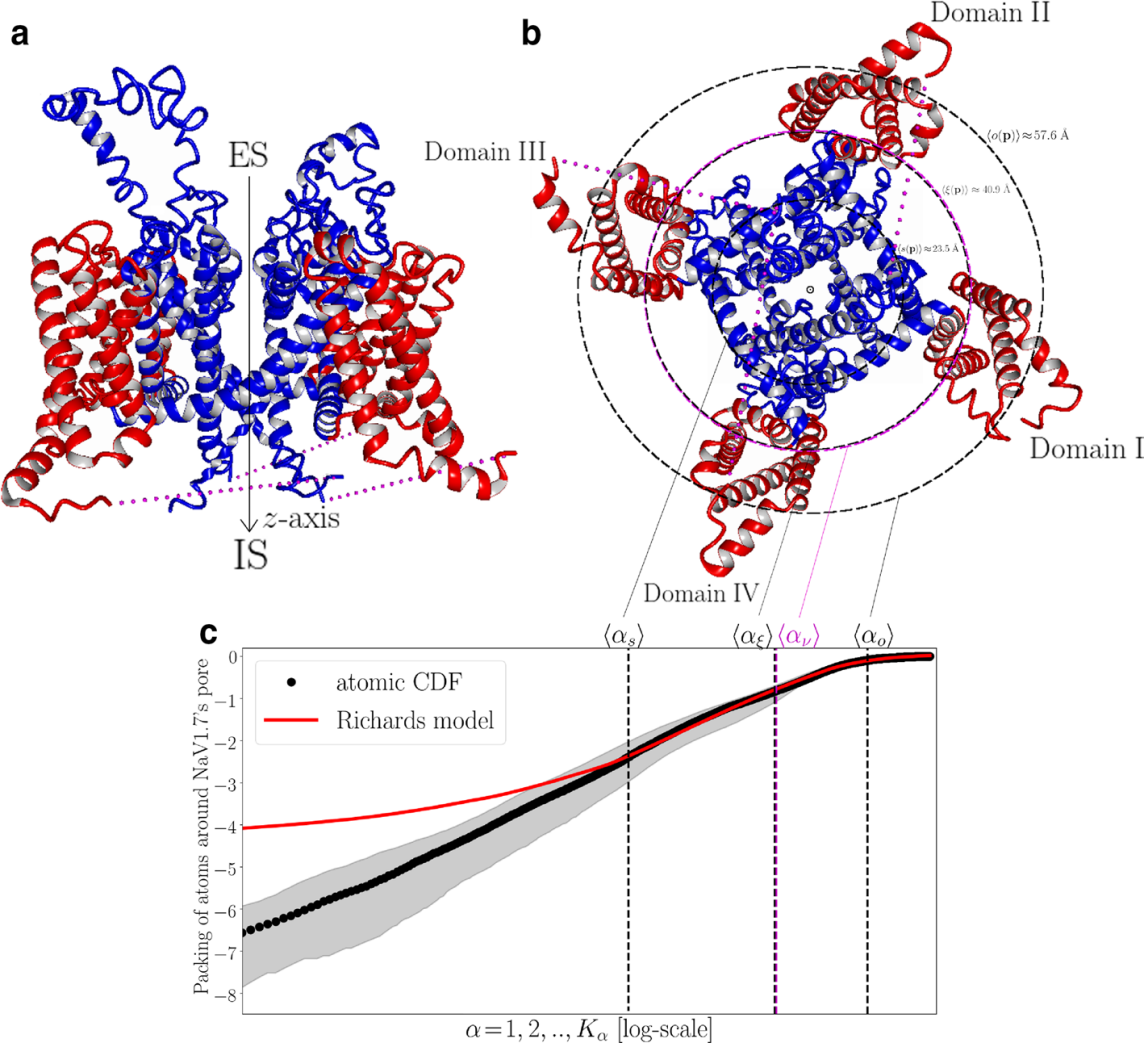
Atom-packing around NaV1.7’s pore. **a** Cartoon illustration of the NaV1.7 structural model (side view). **b** Cartoon illustration of the NaV1.7 structural model (intracellular-to-extracellular view). The atomic environment around every pore point ${\mathbf{p}}\in P$ is partitioned into three consecutive atom-packing domains; a lag domain realized for $l_{\alpha }({\mathbf{p}})\le s({\mathbf{p}})$, an inflection domain consisting of two parts realized for $s({\mathbf{p}})<l_{\alpha }({\mathbf{p}})\le \xi ({\mathbf{p}})$ and $\xi ({\mathbf{p}})<l_{\alpha }({\mathbf{p}})\le o({\mathbf{p}})$, respectively, and an asymptote domain realized for $l_{\alpha }({\mathbf{p}})>o({\mathbf{p}})$ (see “Methods” section). $s({\mathbf{p}})$, $\xi ({\mathbf{p}})$ and $o({\mathbf{p}})$ are represented in **b** in terms of $\langle s({\mathbf{p}}) \rangle$, $\langle \xi ({\mathbf{p}}) \rangle$, and $\langle o({\mathbf{p}}) \rangle$, respectively, roughly indicating the median-statistical value of the radial distance from ${\mathbf{p}}$ at which the transition among subsequent domains takes place. The median-statistical value of the radial distance from ${\mathbf{p}}$ at which the PMs-to-VSs transition, $\langle \nu ({\mathbf{p}}) \rangle$, takes place is also illustrated. Note that $\langle \nu ({\mathbf{p}}) \rangle$ and $\langle \xi ({\mathbf{p}}) \rangle$ are almost indistinguishable, i.e., $\langle \nu ({\mathbf{p}}) \rangle \approx \langle \xi ({\mathbf{p}}) \rangle$. **c** Traces of statistical descriptors of the normalized (with respect to $N_{c}$) atomic CDF, $\langle \bar{N}({\mathbf{p}},l_{\alpha }({\mathbf{p}}))\rangle _{\alpha }$, and of its best-fitted Richards model $\langle n({\mathbf{p}},l_{\alpha }({\mathbf{p}}))\rangle _{\alpha }$ are plotted in log-scale with shaded areas around $\langle N({\mathbf{p}},l_{\alpha }({\mathbf{p}}))\rangle _{\alpha }$ indicative of 95$\%$ confidence intervals. $s({\mathbf{p}})$, $\xi ({\mathbf{p}})$, and $o({\mathbf{p}})$ are represented in **c** in terms of statistical descriptors $\langle \alpha _{s} \rangle$, $\langle \alpha _{\xi } \rangle \approx \langle \alpha _{\nu } \rangle$, and $\langle \alpha _{o} \rangle$, respectively, returning the median-statistical value of corresponding scaling indices $\alpha$. All statistical descriptors correspond to median values and are calculated according to the scheme presented in Additional file 1: S7

In Fig. 1 we demonstrate that atomic CDF around NaV1.7’s pore follows a sigmoid profile which can be adequately described by the Richards model (Additional file 1: S5). The atomic environment around the pore can thus be partitioned into three consecutive atom-packing domains spanning the channel from the inside (i.e., pore-lining environment) to the outside (i.e., voltage-sensing environment) (Fig. 1b, c). The innermost domain corresponds to the lag domain consisting primarily of structural elements lining the pore (approximately 95$\%$ of lag-domain structural components belong to S5–S6 helices). The outermost domain corresponds to the asymptote domain consisting mostly of structural elements drawn from the S1–S4 voltage-sensing helices (approximately 63.3$\%$ of asymptote-domain structural components belong to the S1–S4 helices). In-between the lag and asymptote domain, a structurally-heterogeneous inflection domain is formed consisting of two parts separated by inflection points, $\xi ({\mathbf{p}})$. Structural locations of the inflection points correspond to intra-channel regions where the atomic RDF maximizes and, as demonstrated in Fig. 1a, they closely match the PMs-VSs spatial transition described by $\nu ({\mathbf{p}})$ (see Additional file 1: S6 for calculation of PMs-VSs spatial transition characteristics). Accordingly, $\xi ({\mathbf{p}})$ serves here as a macroscopic boundary line splitting the atomic environment around NaV1.7’s pore into two phases, namely, a pre-inflection phase for $l_{\alpha }({\mathbf{p}})\le \xi ({\mathbf{p}})$ and a post-inflection phase for $l_{\alpha }({\mathbf{p}})>\xi ({\mathbf{p}})$ accounting for atomic sub-environments containing mainly structural components belonging to the PMs and VSs, respectively (Fig. 1b).

Based on the geometrical partition scheme summarized in Fig. 1 we proceeded with mapping of missense *SCN9A*-gene mutations on two-dimensions based on their intra-channel structural locations. For that, we utilized a collection of well-studied GOF NaV1.7 mutations phenotypically related with IEM, SFN and PEPD pain disease (total number of pain-related mutation sites: 36) (Additional file 1: S8). In addition, a collection of neutrals *SCN9A*-gene mutations was introduced consisting of *SCN*9*A*-gene homologous single-nucleotide polymorphisms (hSNPs) and of *SCN*9*A*-gene variants not causing biophysical abnormalities (nBABNs) (imported from [59]), as well as, of a small number of *SCN*9*A*-gene variants previously classified as non-pathogenic based on algorithmic procedures described in [92] (total number of neutral mutation sites: 48) (Additional file 1: S8). Given that neutrals are not expected to associate with pain disease phenotypes, they are less likely to trigger any significant NaV1.7 destabilizations and/or to perturb conserved hydropathic characteristics around NaV1.7’s pore.

**Fig. 2:**
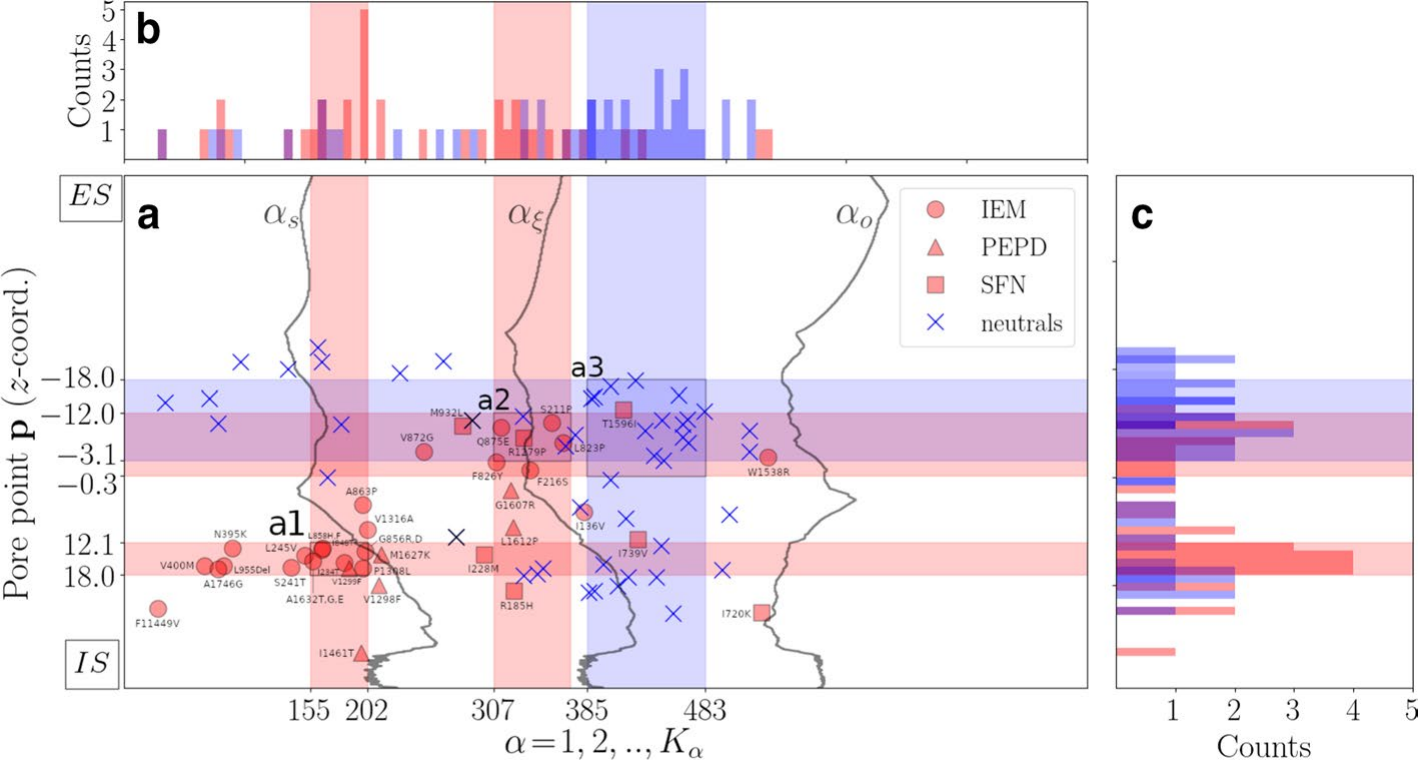
Mapping of missense *SCN9A*-gene mutation sites around NaV1.7’s pore. **a** Mapping of missense *SCN9A*-gene mutation sites for ${\mathbf{p}}\in P$ and $\alpha =1,2,\ldots ,K_{\alpha }=800$. Two sets of missense *SCN9A*-gene mutation sites are employed; a pain-related set containing IEM, PPD and SFN mutation sites, and a neutral set containing mutation sites which are not expected to associate with pain disease phenotypes (Additional file 1: S8). Scaling indices lines $\alpha _{s}$, $\alpha _{\xi }$ and $\alpha _{o}$ highlight the boundaries among consecutive atom-packing domains. Specifically, $\alpha _{s}$ denotes the ending and beginning of the lag and inflection domain, respectively. $\alpha _{o}$ denotes the ending and beginning of the inflection and asymptote domain, respectively. $\alpha _{\xi }$ denotes the location of inflection points and the ending and beginning of the first and second part of the inflection domain, respectively. Labels “a1”, “a2”, and “a3” indicate map areas where the number of mutation sites maximizes, i.e., mutation sites occupancy rates maximize. **a** Map occupancy rate of mutation sites along $\alpha$-direction. **c** Map occupancy rate of mutation sites along ${\mathbf{p}}$-direction. Red- and blue-colored histograms account for map occupancy rates of pain-related, and neutral mutation sites, respectively

The majority (i.e., $54\%$) of pain-related mutation sites are distributed within the inflection domain with a tendency to cluster toward a centrally-located map area along the inflection-points line $\alpha _{\xi }$ (see area “a2” on Fig. 2a in relation to Fig. 2b, c). On the other hand, the majority (i.e., $75\%$) of neutral mutation sites are distributed within the second part of the inflection domain so that their map occupancy rate tends to maximize approximately in the middle of the second part of the inflection domain (see area “a3” on Fig. 2a in relation to Fig. 2b, c). The rest $46\%$ of pain-related mutation sites are found within the lag domain toward the intracellular side of the NaV1.7. In particular, their map occupancy rate maximizes in the vicinity of the boundary line $\alpha _{s}$ toward the IS (see area “a1” on Fig. 2a in relation to Fig. 2b, c). Taken together, these observations indicate that for decreasing molecular scale the probability of a missense *SCN9A*-gene mutation to translate into a pain-related phenotype increases which is of little surprise considering that mutations perturbing NaV1.7’s interior are more likely to perturb tight packing of S5-S6 helices and, consequently, produce electrophysiological alternations.

**Fig. 3:**
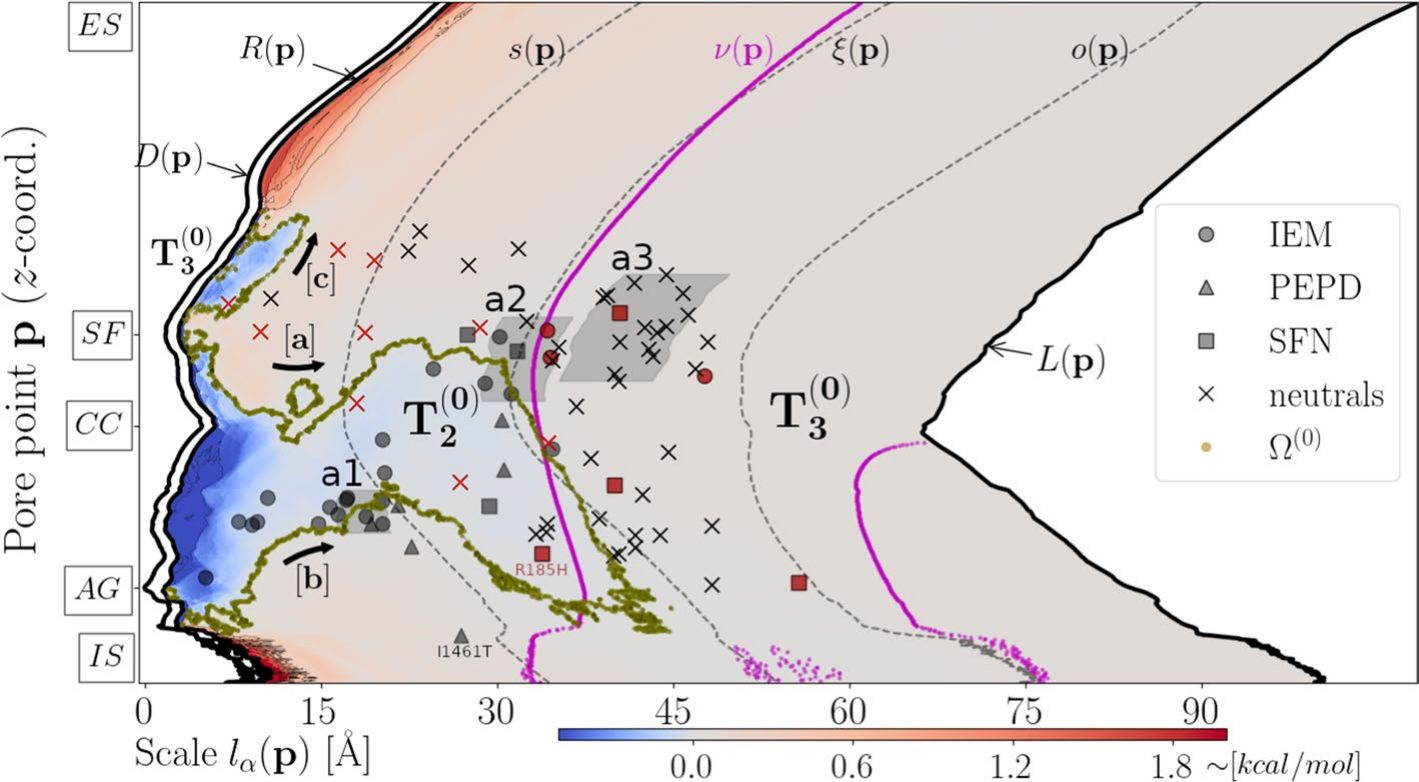
Spatial profile of the hydropathic density around NaV1.7’s pore. Contour map of the hydropathic density pore function, $m^{(0)}({\mathbf{p}},l_{\alpha }({\mathbf{p}}))$, is illustrated for ${\mathbf{p}}\in P$ and $\alpha =1,2,\ldots ,K_{\alpha }=800$. Blue- and red-colored contour domains represent hydrophobic and hydrophilic domains around the pore, respectively. Black lines $R({\mathbf{p}})$, $D({\mathbf{p}})$ and $L({\mathbf{p}})$ indicate geometrical pore characteristics (see “Methods” section). Magenta dashed line $\nu ({\mathbf{p}})$ depicts the scales at which the PMs-VSs spatial transition takes place. Dashed black lines $s({\mathbf{p}})$, $\xi ({\mathbf{p}})$ and $o({\mathbf{p}})$ account for the boundaries among subsequent atom-packing domains (see “Methods” section). Zero-crossing points of $m^{(0)}({\mathbf{p}},l_{\alpha }({\mathbf{p}}))$ collected in $\Omega ^{(0)}$ define HP’s boundary, i.e., the boundary between HP-forming domains $T_{1}^{(0)}$ and $T_{2}^{(0)}$, and hydrophilic domain $T_{3}^{(0)}$ (see Additional file 1: S4 for calculation of zero-crossing points and construction of $\Omega ^{(0)}$). Black arrows $\mathrm{[a]}$, $\mathrm{[b]}$, and $\mathrm{[c]}$ highlight domain boundaries. Two sets of missense *SCN9A*-gene mutation sites are employed; a pain-related set containing IEM, PPD and SFN mutation sites, and a neutral set containing mutation sites which are not expected to associate with pain disease phenotypes (Additional file 1: S8). Mutation sites highlighted in red color correspond to misclassified events (classification criterion; median distance from HP’s boundary (see Additional file 1: S9a)). Grey-shaded areas “a1”, “a2”, and “a3” highlight contour map regions where the number of mutation sites maximizes, i.e., mutation sites occupancy rates maximize. ES, SF, CC, AG, and IS labels mark the locations of the extracellular side, of the selectivity filter, of the central cavity, of the activation gate, and of the intracellular side, respectively

Mapping of hydropathic density profile reveals the formation of a HP lining NaV1.7’s pore (see Fig. 3, blue-colored domains $T^{(0)}_{1}$ and $T^{(0)}_{2}$). Specifically, we demonstrate that the center of the pore is lined by predominantly hydrophobic atomic components expanding toward the IS where occlusion of the pore takes place by the ring of Y405 (DI), F960 (DII) F1449 (DIII) and F1752 (DIV) residues which are known to form the NaV1.7’s activation gate (AG) (see Fig. 3, blue-colored domain $T^{(0)}_{2}$). Hydrophobic expansion toward the ES is disrupted by the weakly-hydrophilic SF so that the pore is lined by a small-sized hydrophobic cluster located in-between the strongly-hydrophilic ES mouth and the weakly-hydrophilic SF (see Fig. 3, blue-colored domain $T^{(0)}_{1}$). Macroscopically, the wide CC translates into a structural contraction event as the outer surface radius is locally minimized so that the channel is split into two funnel-like structural compartments (see Fig. 3, trace of $L({\mathbf{p}})$). Structural combination of the PMs with the VSs results in a hydrophilic atomic environment as indicated by the red-colored $T^{(0)}_{3}$ domain covering the largest map area and incorporating both the SF and the ES mouth, as well as, the IS channel end (see Fig. 3). The SF’s microenvironment is formed by the residues D361 (DI), E930 (DII), K1406 (DIII) and A1698 (DIV) where a bare sodium ion of radius $\sim 1.8$ Å  can exactly fit in (Fig. 3).

Strikingly, approximately $53\%$ of pain-related mutation sites are found within $T^{(0)}_{2}$ with a tendency to cluster along HP’s boundary as map areas “a1” and “a2” occupy contour map regions where the transition from $T^{(0)}_{2}$ to $T^{(0)}_{3}$ takes place (Fig. 3). On the other hand, $10\%$ of neutral mutation sites are located within the $T^{(0)}_{2}$ and only one neutral mutation site found within $T^{(0)}_{1}$, while area “a3” is distributed solely within $T^{(0)}_{3}$ (Fig. 3).

**Fig. 4:**
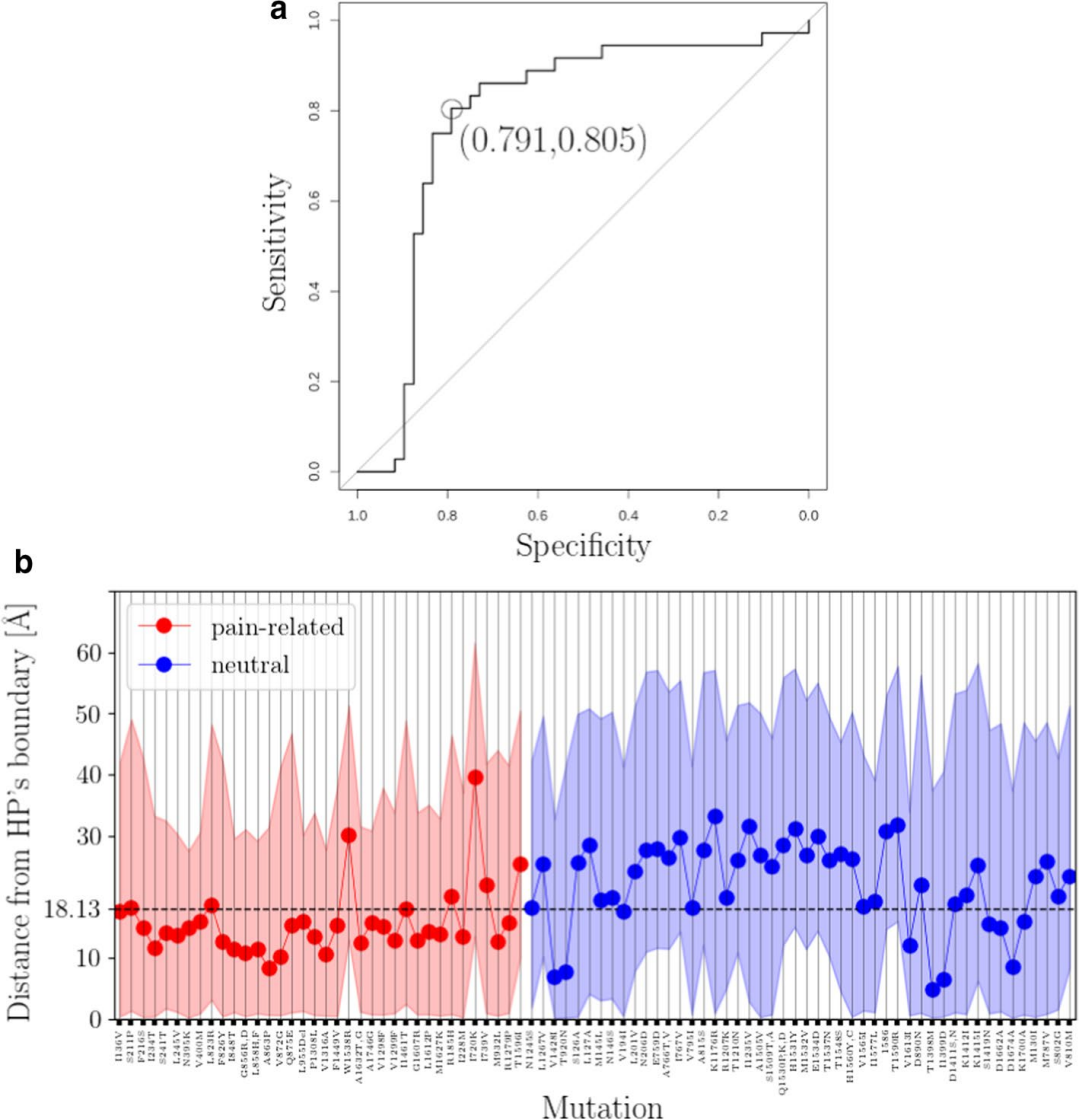
Binary classification of missense *SCN9A*-gene mutation sites based on their median distance from HP’s boundary. **a** ROC-curve plot constructed from data of median distances between mutation sites and HP’s boundary (for construction of data set see Additional file 1: S9a). Optimal threshold value corresponds to specificity and sensitivity values of 0.791 and 0.805, respectively. Area under ROC curve is 0.787. **b** Visualization of ROC curve data. Optimal threshold value 18.13 Å is marked with black dashed line. Shaded area around median distance values indicates the 95$\%$ confidence intervals. ROC curve is constructed in R [73] by using the pROC package [93]. Two sets of missense *SCN9A*-gene mutation sites are employed; a pain-related set containing IEM, PPD and SFN mutation sites, and a neutral set containing mutation sites which are not expected to associate with pain disease phenotypes (Additional file 1: S8)

Given that mutations perturbing an ion channel’s hydrophobic interior properties pose a high risk for ion current alternations [53–56], we hypothesize that mutations occurring at structural locations in proximity to the HP are more likely to be related with GOF pain phenotypes. We tested this hypothesis by statistically approximating the distance between each mutation structural location and HP’s boundary (Additional file 1: S9a) and feeding retrieved median distances into a binary classifier. We achieved to classify correctly 29 (out of 36) and 38 (out of 48) of pain-related and neutral, respectively, mutations correctly with a cut-off median distance of 18.13 Å  (Fig. 4). This translates to an area under receiver operating characteristics (ROC) curve of 0.787 and pain phenotype prediction with specificity of 0.791 and sensitivity of 0.805 (Fig. 4a).

Misclassified pain-related mutations S211P, L823R, W1538R, I720K, I739V and T1596I are found outside of the HP thus not in hydrophobic proximity neither to CC nor to AG (Figs. 3 and 4b). Only a single pain-related misclassification is found within HP, namely, R185H that is located with $T_{2}^{(0)}$ (Figs. 3 and 4b). This striking misclassification is due to the elementary statistical approach adopted for calculating distance scores which fails to fully capture the complex topology of HP-forming $T_{2}^{(0)}$ and $T_{1}^{(0)}$ (Additional file 1: S8). Misclassified neutral mutations V1428I, T920N, V194I, V1613I, T1398N, I1399D, S1419N, D1662A, D1674A and K1700A are found inside the HP, and, more precisely, in proximity to HP’s boundary, with a tendency to cluster around the SF (Figs. 3 and 4b).

**Fig. 5:**
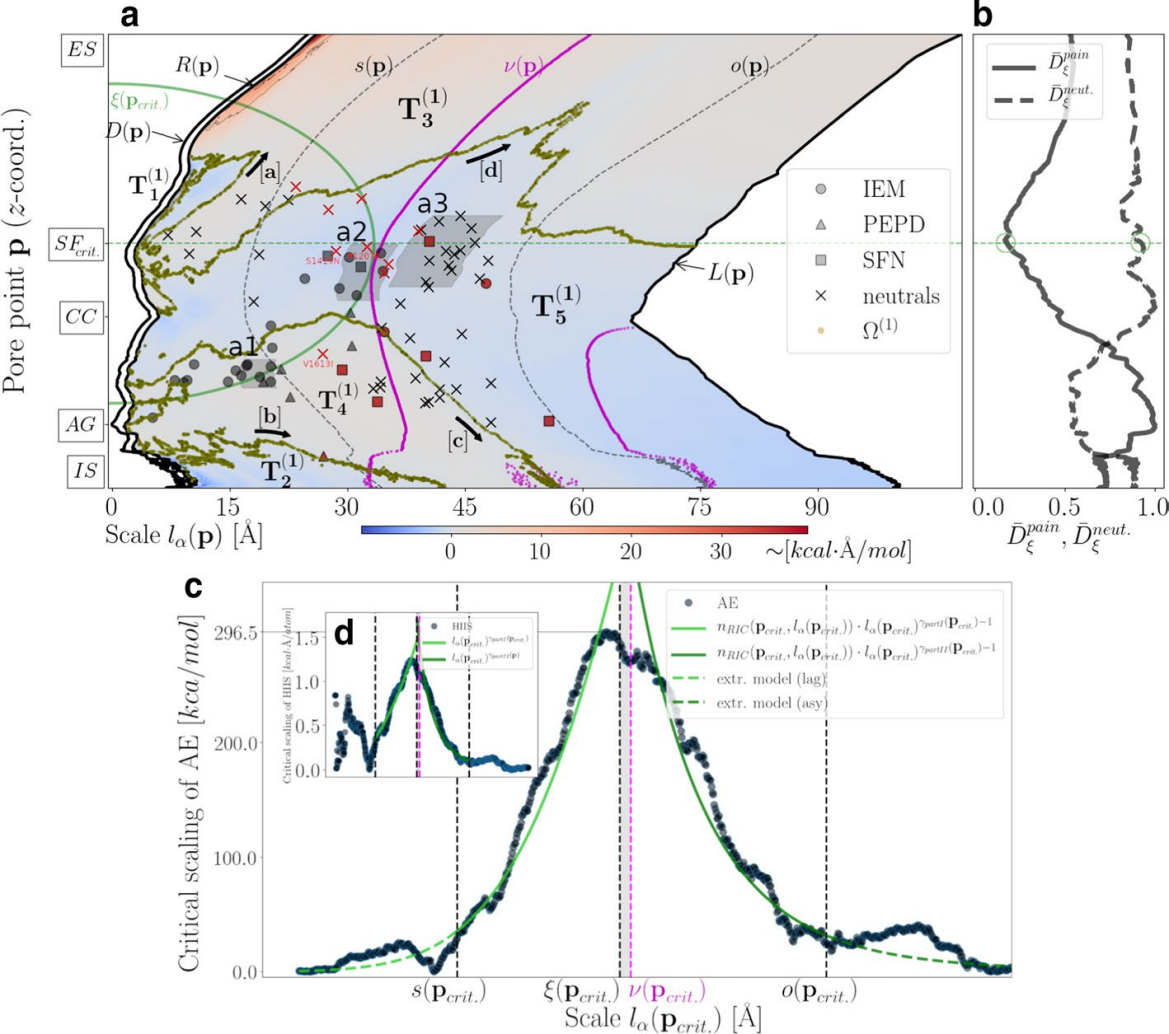
Spatial profile of HIIS axial field component along NaV1.7’s pore. **a** Contour map of HIIS axial field component, $m^{(1)}_{z}({\mathbf{p}},l_{\alpha }({\mathbf{p}}))$, for ${\mathbf{p}}\in P$ and $\alpha =1,2,\ldots ,K_{\alpha }=800$. Blue- and red-colored contour domains represent configurations of ${\vec{\varvec{m}}}^{(1)}_{z}({\mathbf{p}},l_{\alpha }({\mathbf{p}}))$ with orientation “out” and “in”, respectively. Black lines $R({\mathbf{p}})$, $D({\mathbf{p}})$ and $L({\mathbf{p}})$ indicate geometrical pore characteristics (see “Methods” section). Magenta dashed line $\nu ({\mathbf{p}})$ depicts the scales at which the PMs-VSs spatial transition takes place. Dashed black lines $s({\mathbf{p}})$ and $o({\mathbf{p}})$ account for the upper and lower boundary of the lag and asymptote atom-packing domains. Critical radius $\xi ({\mathbf{p}}_{crit.})\approx 33.4$ Å is plotted while inflection points line $\xi ({\mathbf{p}})$ is omitted for clarity. Zero-crossing points of $m^{(1)}_{z}({\mathbf{p}},l_{\alpha }({\mathbf{p}}))$ collected in $\Omega ^{(1)}$ (see Additional file 1: S4) correspond to boundaries among contour domains $T_{1}^{(1)}$, $T_{2}^{(1)}$, $T_{3}^{(1)}$, $T_{4}^{(1)}$ and $T_{5}^{(1)}$. Black arrows $\mathrm{[a]}$, $\mathrm{[b]}$, $\mathrm{[c]}$, $\mathrm{[d]}$, and $\mathrm{[e]}$ are plotted in order to highlight domain boundaries. Two sets of missense *SCN9A*-gene mutation sites are employed; a pain-related set containing IEM, PPD and SFN mutation sites, and a neutral set containing mutation sites which are not expected to associate with pain disease phenotypes (Additional file 1: S8). Mutation sites highlighted with red color correspond to misclassified events (classification criterion; distance from the SF (see Additional file 1: S9b)). Grey-shaded areas “a1”, “a2”, and “a3” highlight contour map regions where the number of mutation sites maximizes, i.e., mutation sites occupancy rates maximize. ES, SF$_{crit}$, CC, AG, and IS labels mark the locations of the extracellular side, of the critical pore point ${\mathbf{p}}_{crit.}$, of the central cavity, of the activation gate, and of the intracellular side, respectively. **b** Traces of normalized (with respect to corresponding maximum values) median distances between pain-related and neutral mutation sites from the critical point, $\xi ({\mathbf{p}})$, represented as $\bar{D}^{pain}_{\xi }=\bar{D}_{\xi ({\mathbf{p}})}(V_{pain})$ and $\bar{D}^{neut.}_{\xi }=\bar{D}_{\xi ({\mathbf{p}})}(V_{neut.})$, respectively, are plotted for ${\mathbf{p}}\in P$ (see Additional file 1: S9b for calculation of $D_{\xi ({\mathbf{p}})}(V_{pain})$ and $D_{\xi ({\mathbf{p}})}(V_{neut.})$). Circles indicate that for ${\mathbf{p}}\approx {\mathbf{p}}_{crit.}$, $\bar{D}^{pain}_{\xi }$ is globally minimized with $\bar{D}^{pain}_{\xi }\approx 0.17$, while $\bar{D}^{neut.}_{\xi }$ exhibits a local maximum with $\bar{D}^{neut.}_{\xi }\approx 0.93$. **c** Trace of $m^{(1)}_{z}({\mathbf{p}},l_{\alpha }({\mathbf{p}}))$ for ${\mathbf{p}}={\mathbf{p}}_{crit.}$ and $\alpha =1,2,\ldots ,K_{\alpha }=800$. Power-law approximations of $m^{(1)}_{z}({\mathbf{p}}_{crit.},l_{\alpha }({\mathbf{p}}_{crit.}))$ described by Eq. r1 are plotted in light and dark green color accounting for the first and second part of the inflection domain, i.e., for $s({\mathbf{p}}_{crit.})<l_{\alpha }({\mathbf{p}}_{crit.})\le \xi ({\mathbf{p}}_{crit.})$ and $\xi ({\mathbf{p}}_{crit.})<l_{\alpha }({\mathbf{p}}_{crit.})\le o({\mathbf{p}}_{crit.})$, respectively. The mean absolute relative fitting errors (MARFEs) of the power-law approximation for the first and second part of the inflection domain are $0.09\pm 0.01$ and $0.15\pm 0.03$, respectively. **d** Trace of AE, $U({\mathbf{p}},l_{\alpha }({\mathbf{p}}))$, for ${\mathbf{p}}={\mathbf{p}}_{crit.}$ and $\alpha =1,2,\ldots ,K_{\alpha }=800$. Modeling approximations of $U({\mathbf{p}}_{crit.},l_{\alpha }({\mathbf{p}}_{crit.}))$ described by Eq. r2 are plotted in light and dark green color accounting for the first and second part of the inflection domain, i.e., for $s({\mathbf{p}}_{crit.})<l_{\alpha }({\mathbf{p}}_{crit.})\le \xi ({\mathbf{p}}_{crit.})$ and $\xi ({\mathbf{p}}_{crit.})<l_{\alpha }({\mathbf{p}}_{crit.})\le o({\mathbf{p}}_{crit.})$, respectively. The MARFEs of the modeling approximation for the first and second part of the inflection domain are $0.11\pm 0.02$ and $0.14\pm 0.03$, respectively. Extrapolation of model approximations toward the lag domain, i.e., for $l_{\alpha }({\mathbf{p}})\le s({\mathbf{p}})$, and toward the asymptote domain, i.e., for $l_{\alpha }({\mathbf{p}})>o({\mathbf{p}})$, are plotted with dashed light and dark green lines, respectively, and result in a MARFE of $6.06\pm 16.0$ and $1.55\pm 6.39$, respectively. Richards model parameters used for modeling AE are $\{A({\mathbf{p}}_{crit.})=1.03,t({\mathbf{p}}_{crit.})=0.03,s({\mathbf{p}}_{crit.})=18.16,\tilde{q}({\mathbf{p}}_{crit.})=0.47\}$

We further investigated the relation between cumulative hydropathic characteristics and mutation sites around NaV1.7’s pore by focusing on the HIIS axial field component illustrated in Fig. 5a. As we demonstrate in in Fig. 5a, HIIS axial field component topology can be summarized into five domains, namely, $T_{1}^{(1)}$, $T_{2}^{(1)}$, $T_{3}^{(1)}$, $T_{4}^{(1)}$ and $T_{5}^{(1)}$ (Fig. 5a). The centrally-located $T_{5}^{(1)}$ domain covers the largest map area and roughly dichotomizes the contour map into two pseudo-symmetric parts, namely, an ES part incorporating $T_{1}^{(1)}$ and $T_{3}^{(1)}$, and an IS part incorporating $T_{2}^{(1)}$ and $T_{4}^{(1)}$. Pain-related mutation sites are solely found within the $T_{4}^{(1)}$ and $T_{5}^{(1)}$ domains with occupancy rates of 58$\%$ and 42$\%$, respectively. On the other hand, neutral sites are found within the $T_{3}^{(1)}$, $T_{4}^{(1)}$ and $T_{5}^{(1)}$ with occupancy rates of 14$\%$, 19$\%$ and 67$\%$, respectively.

In order to decode mutation sites clustering behavior on the contour map of Fig. 5a we adopted a phenomenological approach that presumes the existence of a critical point, $\xi ({\mathbf{p}}_{crit.})$, associated with the spatial organization of HIs around the SF [63, 64, 69] with ${\mathbf{p}}_{crit.}$ being a critical SF pore point coordinate (Additional file 1: S10). A crucial result that motivated us to adopt such an approach is that pain-related mutation sites are attracted toward the critical point in sheer contrast to neutral mutation sites which are repelled from it (Fig. 5b). We refer to this phenomenon with the term critical clustering. Geometrically, the formation of the critical mutation-sites cluster reflects the tendency of structural locations of pain-related mutations to minimize their distance from the surface of the sphere of radius $\xi ({\mathbf{p}}_{crit.})\approx 33.4$ Å  around the SF; intuitive graphical representation of this phenomenon is provided in Fig. 5a where we show that “hot” areas I and II intersect with the green radius line $\xi ({\mathbf{p}}_{crit.})$ representing critical sphere’s surface.

The scaling behavior of HIIS axial field component around ${\mathbf{p}}_{crit.}$ is adequately described in terms of the power-law scheme

r1 $$\begin{aligned} m^{(1)}_{z}({\mathbf{p}}_{crit.},l_{\alpha }({\mathbf{p}}_{crit.})) \sim {\left\{ \begin{array}{ll} l_{\alpha }({\mathbf{p}}_{crit.})^{\gamma _{partI}({\mathbf{p}}_{crit.})} {\text{ for }}\,\,s({\mathbf{p}}_{crit.})<l_{\alpha }({\mathbf{p}}_{crit.})\le \xi ({\mathbf{p}}_{crit.})\\ l_{\alpha }({\mathbf{p}}_{crit.})^{\gamma _{partII}({\mathbf{p}}_{crit.})} {\text{ for }}\,\,\xi ({\mathbf{p}}_{crit.})<l_{\alpha }({\mathbf{p}}_{crit.})\le o({\mathbf{p}}_{crit.}) \end{array}\right. } \end{aligned}$$

accounting for a HIs-network expansion and contraction within the pre- and post-inflection phase intervals $s({\mathbf{p}}_{crit.})<l_{\alpha }({\mathbf{p}}_{crit.})\le \xi ({\mathbf{p}}_{crit.})$ and $\xi ({\mathbf{p}}_{crit.})<l_{\alpha }({\mathbf{p}}_{crit.})\le o({\mathbf{p}}_{crit.})$ with rates of $\gamma _{partI}({\mathbf{p}}_{crit.})=2.27\pm 0.18$ and $\gamma _{partII}({\mathbf{p}}_{crit.})=-5.18\pm 1.02$, respectively (Fig. 5c, and see also Additional file 1: S10). On the left of the interval $\xi ({\mathbf{p}}_{crit.})<l_{\alpha }({\mathbf{p}}_{crit.})\le \nu ({\mathbf{p}}_{crit.})$, both, the range and intensity of HIIS axial field component maximize as the HIs-network configuration exceeds its critical size marking the transition from the pre-inflection phase toward the post-inflection phase [69]. The energy levels associated with this phase transition are given by

r2 $$\begin{aligned} U({\mathbf{p}}_{crit.},l_{\alpha }({\mathbf{p}}_{crit.})) \sim {\left\{ \begin{array}{ll} N({\mathbf{p}}_{crit.},l_{\alpha }({\mathbf{p}}_{crit.}))\cdot l_{\alpha }({\mathbf{p}}_{crit.})^{\gamma _{partI}({\mathbf{p}}_{crit.})-1} {\text{ for }}\,\,s({\mathbf{p}}_{crit.})<l_{\alpha }({\mathbf{p}}_{crit.})\le \xi ({\mathbf{p}}_{crit.})\\ N({\mathbf{p}}_{crit.},l_{\alpha }({\mathbf{p}}_{crit.})\cdot l_{\alpha }({\mathbf{p}}_{crit.})^{\gamma _{partII}({\mathbf{p}}_{crit.})-1} {\text{ for }}\,\,\xi ({\mathbf{p}}_{crit.})<l_{\alpha }({\mathbf{p}}_{crit.})\le o({\mathbf{p}}_{crit.}) \end{array}\right. } \end{aligned}$$

where $N({\mathbf{p}}_{crit.},l_{\alpha }({\mathbf{p}}_{crit.})$ can be replaced with its best-fitted Richards model, $n_{ric}({\mathbf{p}}_{crit.},l_{\alpha }({\mathbf{p}}_{crit.}))$ (see caption of Fig. 5 for Richards model parameters), providing with an estimation of the atom-packing energy (AE) (Fig. 5c). Similarly to the NaVAb case [69], AE maximization occurs in the vicinity of the narrow interval $[\xi ({\mathbf{p}}_{crit.}),\nu ({\mathbf{p}}_{crit.})]$ so that energetic coupling of the PMs with the VSs is dictated by the phase transition (Fig. 5c, d).

Equation r1 indicates that interatomic HIs law is robust to microscopic modifications of the atomic structure, e.g., addition, removal or deletion of a small number of atoms occurring due to a mutation-induced perturbation of $N({\mathbf{p}}_{crit.},l_{\alpha }({\mathbf{p}}_{crit.}))$.^1^ This happens however at the cost of re-tuning HIcIS and, hence, also AE, that in the case of small-amplitude perturbations of $N({\mathbf{p}}_{crit.},l_{\alpha }({\mathbf{p}}_{crit.}))$, are expected to be up- and down-regulated toward and away from the critical point, respectively, in a power-law fashion described by r2 (Fig. 5c). Mutation-induced perturbations propagating throughout the structure are thus expected to be amplified in the vicinity of the critical point while, on the other hand, to be damped out toward the interior (i.e., toward the HP and the SF) and toward channel exterior bounded by outer pore surface radius. Given that mutations occurring in the structural proximity of the SF are highly likely to have a deleterious LOF effect [94], observed damping-out mechanism might act as a shield protecting SF’s biological machinery from mutations occurring within the pre-inflection phase. On the other hand, mutations occurring in the post-inflection phase are unlikely to perturb the SF as they have to overcome a large energy barrier in order to reach channel interior. We thus hypothesize that critical clustering of pain-related mutation sites might actually reflect a trade-off between the two extremes; a destructive destabilization and an insignificant one.

**Fig. 6:**
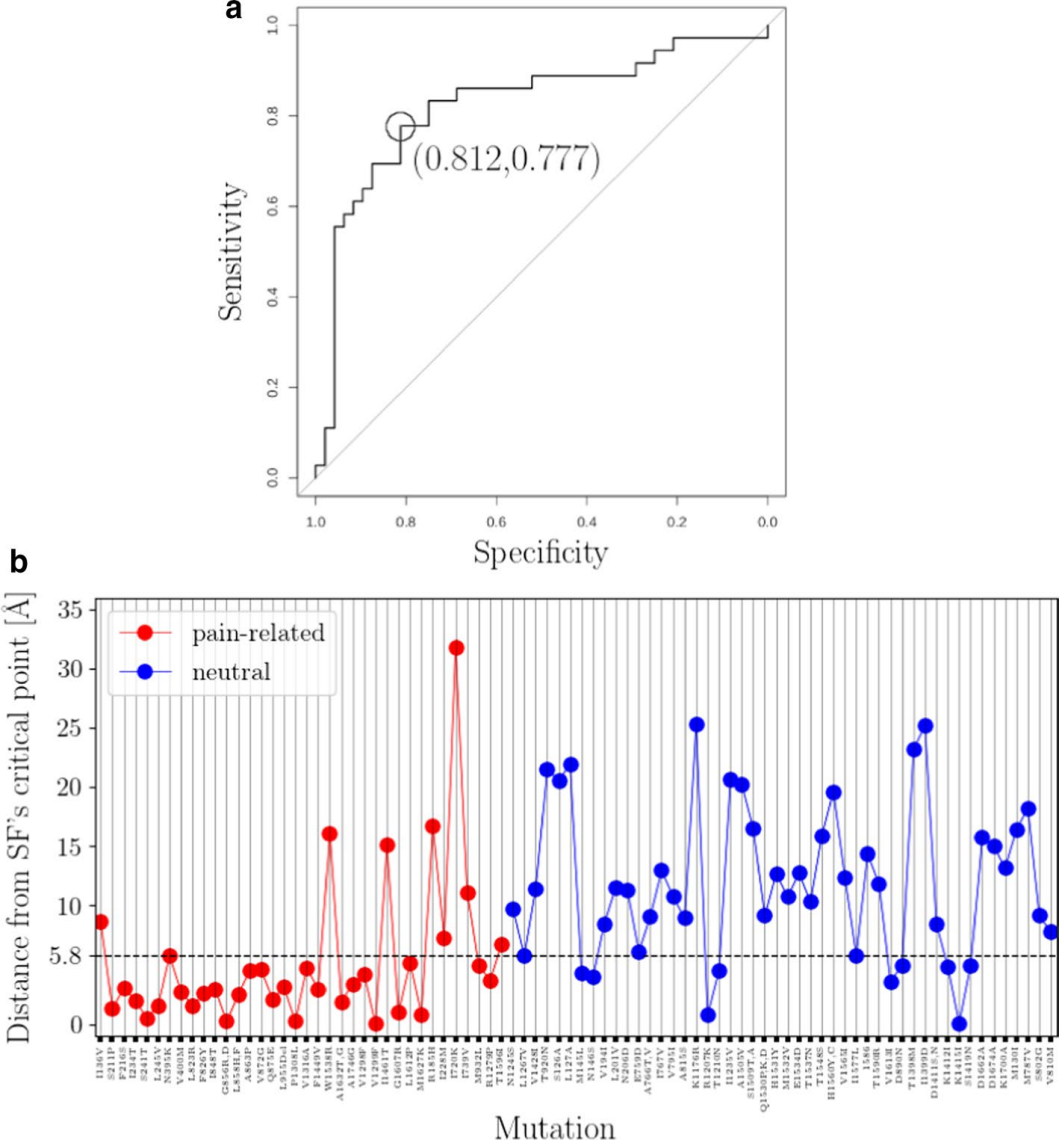
Binary classification of missense *SCN9A*-gene mutation sites based on their distance from SF’s critical point. **a** ROC curve constructed from data of distances between mutation sites and SF’s critical-point (for construction of data set see Additional file 1: S9b). Optimal threshold value $\sim$5.8 Å corresponds to specificity and sensitivity values of 0.812 and 0.777, respectively. Area under ROC curve is 0.824. **b** Visualization of ROC curve data. Optimal threshold value $\sim$5.8 Å is marked with black dashed line. ROC curve is constructed in R [73] by using the pROC package [93]. Two sets of missense *SCN9A*-gene mutation sites are employed; a pain-related set containing IEM, PPD and SFN mutation sites, and a neutral set containing mutation sites which are not expected to associate with pain disease phenotypes (Additional file 1: S8)

We tested the critical-clustering hypothesis by calculating the distance of each mutation site from SF’s critical point (Additional file 1: S9b) and feeding retrieved distances into a binary classifier. We achieved to classify correctly 28 (out of 36) and 39 (out of 48) of pain-related and neutral mutation sites correctly with a cut-off distance of $\sim$5.8 Å . This translates to an area under receiver operating characteristics (ROC) curve of 0.824 and pain phenotype prediction with specificity of 0.812 and sensitivity of 0.777 (Fig. 6a). Intuitive geometrical depiction of this result requires to think of a “hot” spherical shell squeezed between the spheres of radii $\sim \xi ({\mathbf{p}}_{crit.})+5.8$ Å  and $\sim \xi ({\mathbf{p}}_{crit.})- 5.8$ Å  centered at ${\mathbf{p}}_{crit.}$ incorporating areas “a1” and “a2” thus containing the majority of pain-related mutation sites. This tendency can be deduced from Fig. 5 where we can see that correctly-classified pain-related and neutral mutation sites tend to minimize and maximize, respectively, their distance from the critical radius $\xi ({\mathbf{p}}_{crit.})$. The opposite holds for misclassified mutation sites. Note however that due to the pore points offset, distances of sites from $\xi ({\mathbf{p}}_{crit.})$ line on Fig. 5 are not equal with the distances of their structural locations from the surface of the sphere of $\xi ({\mathbf{p}})$ (discrepancies are of order 3.13±4.63 Å). Misclassified pain-related mutations are I136V, W1538R, I1461T, R185H, I228M, I720K, I739V and T1596I indicating that sensitivity output is qualitatively similar to the HP-based classification attempt. On the other hand, quality of specificity differs significantly among classification attempts as critical-point distance criterion misclassified neutrals M145L, M146S, R1207K, T1210N, V1613I, D890N, K1412I, K1415I and S1419N are clustering within the “hot” spherical shell in proximity to HP’s boundary.

**Fig. 7:**
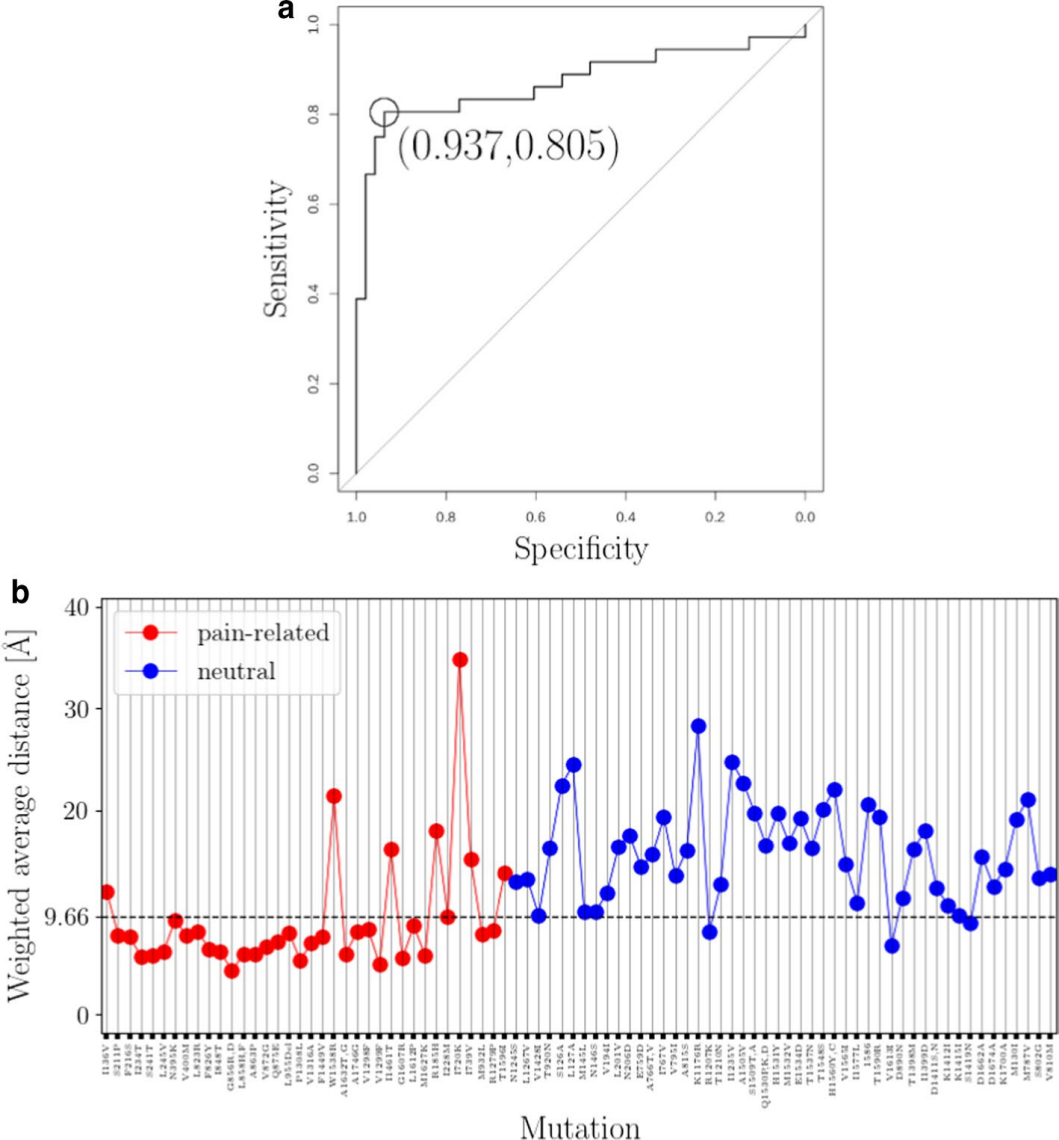
Binary classification of missense *SCN9A*-gene mutation sites based on a weighted distance average. **a** ROC curve constructed from data of distances between mutation sites and the weighted combination of SF’s critical-point and HP’s boundary (for construction of data set see Additional file 1: S11). Optimal threshold value $\sim$9.6 Å corresponds to specificity and sensitivity values of 0.805 and 0.937, respectively. Area under ROC curve is 0.872. **b** Visualization of ROC curve data. Optimal threshold value $\sim$9.6 Å is marked with black dashed line. ROC curve is constructed in R [73] by using the pROC package [93]. Two sets of missense *SCN9A*-gene mutation sites are employed; a pain-related set containing IEM, PPD and SFN mutation sites, and a neutral set containing mutation sites which are not expected to associate with pain disease phenotypes (Additional file 1: S8)

Finally, in order to harvest the classification power of both predictors, we linearly combined distance metrics by calculating a weighted distance average (Additional file 1: S11). The weighted distance average achieved to classify correctly 29 (out of 36) pain-related mutations and 45 (out of 48) neutrals, i.e., sensitivity = 0.805, specificity = 0.937, area under ROC curve = 0.872 (Fig. 7a). The threshold weighted distance value is $\sim$9.6 Å and it indicates which mutation sites are found in proximity to SF’s critical point and HP’s boundary.

The relatively-low sensitivity of the weighted distance average is not surprising if we consider that both classifications attempts failed in correctly classifying pain-related mutation sites found far away from the HP and from the SF; misclassified pain-related mutation sites are I136V, W1538R, I1461T, R185H, I720K, I739V and T1596I and all of them are found within the post-inflection phase with the exception of I1461T which is located within the lag domain but still far away from the HP and from the SF (Figs. 3, 5a and 7). On the other hand, misclassified neutrals R1207K, V1613I and S1419N occupy “hot” spots located in proximity to SF’s critical point and HP’s boundary (Figs. 3, 5a and 7).

## Discussion and concluding remarks

Criticality hypothesis in biology aims at explaining how emergence of power-laws increases biological system’s robustness and efficiency hand-in-hand with evolution. Empirical evidence for complex biological systems operating near critical points include cases of gene expression [95], DNA sequences [96], protein structures [63–66], cell growth [97] and neuronal dynamics underlying brain activity [98]. In practice, criticality implies that system dynamics are delicately balanced between an ordered state where perturbations are damped-out and a disordered state where perturbations are amplified. Consequences of critical dynamics are associated with optimal information processing [99], enhanced network stability [100] and maximal sensitivity to external stimuli [101].

In this work, instead of trying to predict the effect of missense *SCN9A*-gene mutations via comparing mutant NaV1.7 structures in silico, we extracted hydropathic features of the wild-type atomic environment encoding NaV1.7’s response to mutation-induced variations. Stated differently, we hypothesized that some regions of the atomic environment around NaV1.7’s pore exhibit higher sensitivity to mutation-induced perturbations due to the long-range nature of HIs guaranteeing their stability; a hallmark of SOC is that avalanche-like perturbing effects are amplified and fast-spreading throughout critical network locations [102]. To test this hypothesis we mapped mutation structural locations on their corresponding mutation sites and probed topological and scaling hydropathic characteristics of the atomic bulk around the pore. Importantly, this is possible due to the relatively-large number of pain-related mutations providing with the opportunity of structure-based mutation statistics and, consequently, identification of densely-populated (by mutation sites) structural domains.

We employed a closed-state structural model of the NaV1.7 that is constructed in silico via homology modeling procedures based on the pre-open NaVAb template (see “Methods” section). By doing so, we sought to initiate our investigations from a well-studied and precisely-engineered protonated NaV1.7 structure that has previously provided with clinically-relevant observations. The fact that the structural model in-use represents a protonated state of the NaV1.7 molecule is crucial here as cumulative hydropathicity-property moments calculations take explicitly into account hydrogen atoms. Biophysical significance of the structural model in-use was first experimentally validated in [7] where structure-based analysis successfully predicted that the IEM-related V400M and S241T mutations are energetically coupled and, hence, both exhibiting pharmacoresponsiveness to carbamazepine. The same structural model has later also been used in [30, 58] in order to explain mutation-induced electrophysiologal alternations in relation to atomic-level NaV1.7 structural changes. The main structural difference between the model in use here and the 6J8J NaV1.7 [82] structure is that the latter contains the complete DIII-DIV intracellular linker which is a hotspot for PEPD mutations. Thus working with the 6J8J NaV1.7 structure will allow to investigate two more mutation cases, namely, the PEPD-related mutations F1462V [103] and T1464I [15]. Given however that the present scheme fails in classifying correctly the PEPD-related I1461T mutation, it is rather unlikely that it will succeed with F1462V or T1464I. The reason for this misclassification is that the IS end of the channel, where the DIII-DIV linker helix is found, is predominantly hydrophilic and far away from the SF (see Figs. 3 and 5a).

The starting point of the presented procedures was the approximation of the atomic cumulative distribution function around NaV1.7’s pore demonstrating that packing of atoms follows a sigmoid pattern. The generality of the Richards model was found to be adequate for this modeling purpose verifying the sigmoid CDF hypothesis and, consequently, revealing a biphasic spatial organization of the atomic environment around the pore dictated by the spatial transition from the PM from the VSs. We showed that the pore is lined by a HP dominating within channel interior and that HIs stabilizing atom-packing around the SF are critically tuned with respect to the local inflection points. This NaV1.7 feature is shared with its evolutionary-ancestor, namely, with the pre-open NaVAb channel, suggesting that location and nature of HIs critical tuning might be conserved from NaVChs of bacterial homomers to NaVChs of mammalian heteromers [69].

Pain-related mutations tend to occupy structural locations in proximity to HP’s boundary while maintaining a critical HIs-distance from the SF. Geometrically, this result indicates that the majority of pain-related mutations are found within a spherical shell around the SF incorporating parts of the HP. What might be the evolutionary principle underlying this non-random mutation distribution around NaV1.7’s pore? Given that the hydrophilic DEKA SF sequence is conserved among human and non-human NaVCh templates [82], we propose that occurrence of mutations at critical hydropathic-interactions distance from the SF might reflect an evolutionary trade-off between potentially-deleterious destabilizations occurring too close to the SF, and insignificant polymorphisms occurring far away from it. According to this rationale, mutations occupying critical hydropathic-interactions network locations lead to a GOF effect by increasing channel’s configurational space and, consequently, expanding physiological range of ion currents, while not risking structure deletion or severe destabilizations that can induce a LOF effect [94].

Misclassification of seven pain-related events found within the post-inflection phase (namely, of I136V, W1538R, I1461T, R185H, I720K, I739V and T1596I) suggests that the destabilizing mutation effect within the post-inflection phase and, specifically, within the VSs needs to be locally investigated. In particular, misclassified pain-related events are likely to perturb local features of the VSs which are however crucial for physiological gating behavior. It might therefore be useful for future studies to consider a decoupling of the PM from the VSs in order to focus solely on the cumulative hydropathic topology and HIs-networking within the VSs. Alternatively, considering biophysical characteristics of substituted amino acids (e.g., size, charge, degree of conservation) might also contribute in improving classification accuracy as it would provide a more detailed picture of the mutation effect.

Admittedly, a limitation of this study is the small (from a statistics point of view) number of available mutation sites. To resolve this issue and provide with stronger statistical validation, we may consider in future studies to increase number of neutral and pain-related mutations, for example, by introducing NaV1.7 variants found in the Genome Aggregation Database (gnomAD) [104]. A methodological weakness is that we neglected radial hydropathic effects. In particular, even if the amplitude of the HIIS radial field component is decreasing (in comparison to the amplitude of the HIIS axial field component) for increasing molecular scale, its contribution cannot be neglected for interactions between penetrating ions species and pore walls.

In summary, our findings suggest that pathophysiological evaluation of mutation sites with respect to cumulative NaV1.7 hydropathic properties can be performed with negligible computational effort and similar or even higher accuracy to [59] (reported accuracy: 0.81) but also to the more recent study of [61] where a MLE computational pipeline was employed (reported accuracy on the human NaV1.7 template: 63.5%). Given that the formation of a narrow and hydrophilic SF followed by a wide and hydrophobic CC is a widely conserved pore-architectural motif, our observations might be relevant for other voltage-gated channel species as well. Crucially, hydropathicity-property has been previously recognized as a key-marker for predicting functional outcome of genetic defects not only in NaVChs, but also in voltage-gated calcium [94] and potassium channels [105]. Finally, in an era where MLE pipelines become increasingly popular, the phenomenological framework curated in this study could provide a phenomenological basis for biophysical interpretation of MLE-retrieved predictions regarding NaVChs pathophysiological characterization and help in tracing them back on the atomic structure in a site-specific manner as recently attempted in [106].

## Supplementary Information

**Additional file 1:** Supplementary information.

## Data Availability

Data sharing is not applicable to this article as no datasets were generated or analyzed during the current study. The 3D structural model of the NaV1.7 channel is available from the authors with permission of YY and SGW.

## Footnotes

1 The average number of atoms per amino acid is 19.2 corresponding to a minor fraction of $N_{c}=18567$ atoms composing the NaV1.7 structural model in use. Hence, a missense *SCN*9*A*-gene mutation resulting in the substitution or deletion of a single amino acid is expected to induce a perturbation of the form $N({\mathbf{p}}_{crit.},l_{\alpha }({\mathbf{p}}_{crit.}))\rightarrow N({\mathbf{p}}_{crit.},l_{\alpha }({\mathbf{p}}_{crit.}))+\epsilon ({\mathbf{p}}_{crit.},l_{\alpha }({\mathbf{p}}_{crit.}))$, with $\epsilon ({\mathbf{p}}_{crit.},l_{\alpha }({\mathbf{p}}_{crit.})$ being a small-amplitude perturbation source describing addition and/or removal of a minor number of atoms across different channel scales. Then, HIcIS is regulated according to $h^{(1)}_{z}({\mathbf{p}}_{crit.},l_{\alpha }({\mathbf{p}}_{crit.}))+ \zeta ({\mathbf{p}}_{crit.},l_{\alpha }({\mathbf{p}}_{crit.}))\sim l_{\alpha }({\mathbf{p}}_{crit.})^{\gamma ({\mathbf{p}}_{crit.})}\cdot \big ( N({\mathbf{p}}_{crit.},l_{\alpha }({\mathbf{p}}_{crit.}))+\epsilon ({\mathbf{p}}_{crit.},l_{\alpha }({\mathbf{p}}_{crit.})\big )$ with $\gamma ({\mathbf{p}}_{crit.})=\gamma _{partI}({\mathbf{p}}_{crit.})\text { for }\,\,s({\mathbf{p}}_{crit.})<l_{\alpha }({\mathbf{p}}_{crit.})\le \xi ({\mathbf{p}}_{crit.})$ and $\gamma ({\mathbf{p}}_{crit.})=\gamma _{partII}({\mathbf{p}}_{crit.})\text { for }\,\,\xi ({\mathbf{p}}_{crit.})<l_{\alpha }({\mathbf{p}}_{crit.})\le o({\mathbf{p}}_{crit.})$ so that HIIS axial field component described by r1 remains intact around the critical point.
