# Supplementary material for: Hydropathicity-based prediction of pain-causing NaV1.7 variants
Source: BMC Bioinformatics. 2021 Apr 23;22:212. doi: 10.1186/s12859-021-04119-2 (PMC8063372; doi:10.1186/s12859-021-04119-2)
Supplement: Supplementary file 1 — Additional file 1: Supplementary information. [file 12859_2021_4119_MOESM1_ESM.pdf]

Hydropathicity-based prediction of pain-causing NaV1.7 variants

Makros N. Xenakis[a,b,*], Dimos Kapetis[c], Yang Yang[d,e], Monique M. Gerrits[f], Jordi Heijman[g], Stephen G. Waxman[h,i], Giuseppe Lauria[c,j], Catharina G. Faber[k], Ronald L. Westra[l], Patrick J. Lindsey[a,m], Hubert J. Smeets[a,b]

[a]*Department of Toxicogenomics, Section Clinical Genomics, Maastricht University, PO Box 616, 6200 MD Maastricht, the Netherlands*
[b]*Research School for Mental Health and Neuroscience (MHeNS), Maastricht University, PO Box 616, 6200 MD Maastricht, The Netherlands*
[c]*Neuroalgology Unit, Fondazione IRCCS Istituto Neurologico "Carlo Besta", via Celoria 11, 20133 Milan, Italy*
[d]*Department of Medicinal Chemistry and Molecular Pharmacology, Purdue University College of Pharmacy, West Lafayette, IN, 47907, USA*
[e]*Purdue Institute for Integrative Neuroscience, West Lafayette, IN 47907, USA*
[f]*Department of Clinical Genetics, Maastricht University Medical Center, PO box 5800, 6202 AZ, Maastricht, the Netherlands*
[g]*Department of Cardiology, CARIM School for Cardiovascular Diseases, Maastricht University, PO Box 616, 6200 MD Maastricht, The Netherlands*
[h]*Department of Neurology and Center for Neuroscience and Regeneration Research, Yale University School of Medicine, New Haven, CT 06510, USA.*
[i]*Rehabilitation Research Center, Veterans Affairs Connecticut Healthcare System, West Haven, CT 06516, USA.*
[j]*Department of Biomedical and Clinical Sciences "Luigi Sacco", University of Milan, via G.B. Grassi 74, 20157 Milan, Italy*
[k]*Department of Neurology, Maastricht University Medical Center, PO Box 5800, 6202 AZ Maastricht, The Netherlands*
[l]*Department of Data Science and Knowledge Engineering, Maastricht University, PO Box 616, 6200 MD Maastricht, the Netherlands*
[m]*Research School for Oncology and Developmental Biology (GROW), Maastricht University, PO Box 616, 6200 MD Maastricht, the Netherlands*

Supplementary Material

Section S1. Comparison of NaV1.7 structures

The NaV1.7 structural model in use throughout this study shares more than 98% sequence similarity with the recently resolved via cryo-EM NaV1.7

*Correspondence and requests for materials should be addressed to M.N.X. E-mail: mrksxenakis@gmail.com

structure [PDB code: 6J8J] [1]. Superposition of the two heavy-atom structures within Yasara software (Yet Another Scientific Artificial Reality Application, `www.yasara.org`) by using the Mustang algorithm [2] reported on a root mean square deviation (RMSD) of 2.255 Å over 552 aligned residues with 86.05% sequence identity. Relaxing alignment accuracy by using the local Smith&Waterman algorithm [3] results in a RMSD of 7.33 Å over 1111 aligned residues with 99.91% sequence identity. Given that the 6J8J NaV1.7 structure is provided at a resolution of 3.2 Å, results of the Mustang and local Smith&Waterman algorithms suggest that the two structures are closely-related, potentially, both capturing the NaV1.7 at an inactivated conformation.

The main difference between the two structures is the sequence part F1462:K1484 (shown in blue color in Figure S1) which belongs to the DIII-DIV intracellular linker formed via helical arrangement of G1458:N1491 residues. The DIII-DIV intracellular linker is predominantly hydrophilic with a hydropathicity score of $\sim$1.92 kcal/residue as it contains only twelve hydrophobic residues (percentage of hydrophobic residues; 35%), namely, residues I1461, F1462, M1463, Y1470, Y1471, M1474, L1477, P1482, P1485, I1486, P1487, and P1489 (note that, in accordance with the Main Text, hydropathicity of residues is extracted from the Kapcha-Rochky hydropathic scale [4]).

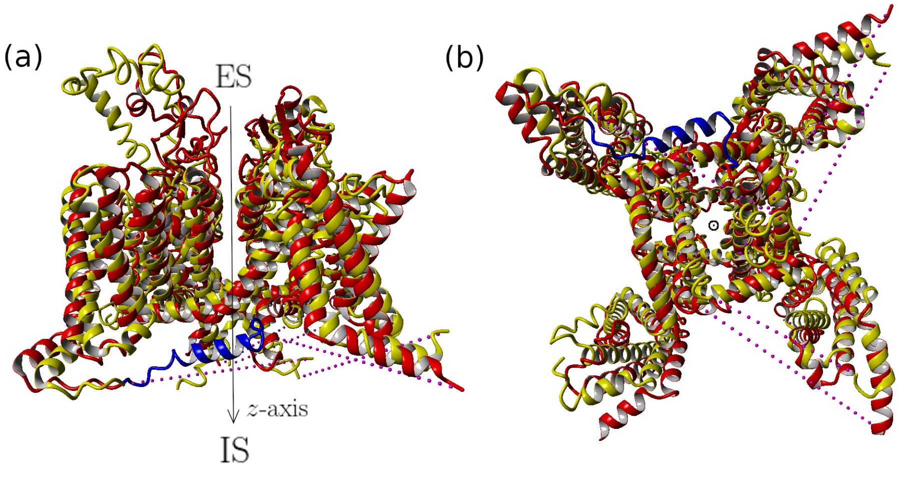

Figure S1: **Superposition of heavy-atom NaV1.7 structures. (a)**, Cartoon illustration of a structural superposition of the 6J8J NaV1.7 structure [1] upon the NaV1.7 structural model in use throughout this study (for model derivation and refinement see Main Text, Methods) (side view). **(b)**, Top-view cartoon illustration of the same structural superposition (intracellular-to-extracellular view). Structural alignment implemented by Mustang algorithm [2]. The 6J8J NaV1.7 structure is shown in red color. The NaV1.7 structural model in use throughout this study is illustrated in yellow color. The residue sequence F1462:K1484 which is part of the DIII-DIV intracellular linker is highlighted in blue color in the 6J8J NaV1.7 structure.

Section S2. Navigating through NaV1.7's pore

The HOLE routine was called $n = 100$ times. During each call, HOLE control parameters are fixed but the searching path is randomly reset according to the rule RASEED = $i \cdot 1000$ with $i = 1, 2.., n$ (see Table S1). At the end of each call we obtain a set of points navigating through the pore where each navigation point, $\mathbf{q}_i = (\mathrm{q}_{x,i}, \mathrm{q}_{y,i}, \mathrm{q}_{z,i}) \in Q$, is located on a membrane-parallel plane. The distance between subsequent membrane-parallel planes is set to 0.1 Å (see Table S1). After the completion of all the calls, we collected all the membrane-parallel planes with each of them containing n navigation points and, under the Gaussian assumption, the geometrical location of a pore point was approximated by

$$\mathbf{p} = (\frac{1}{n}\sum_{i=1}^{n} \mathrm{q}_{x,i}, \frac{1}{n}\sum_{i=1}^{n} \mathrm{q}_{y,i}, \frac{1}{n}\sum_{i=1}^{n} \mathrm{q}_{z,i}) \in P \tag{S1}$$

where P contains $N_\mathrm{p} = 920$ pore points (note that N_p depends on the choice of HOLE parameters (see Table S1)). Due to the skewness of the pore, x and y coordinates of pore points are non-zero. Hence, pore points are radially displaced from the z-axis with an average offset of 3.13±4.63 Å.

Parameter	Value
CVECT	0.0 0.0 1.0
CPOINT	$\mathbf{e}$
SAMPLE	0.1
MCDISP	0.03
MCKT	0.05
MCSTEP	1000
ENDRAD	25.
RASEED	$i\cdot 1000$

Table S1: **HOLE routine parameters.** Parameter CVECT specifies a vector which is aligned with the direction of the channel's pore. Parameter CPOINT corresponds to the atomic center $\mathbf{e}=(\mathrm{e}_x, \mathrm{e}_y, \mathrm{e}_z)$ of the NaV1.7 structure (see Methods). SAMPLE parameter determines the distance between subsequent membrane-parallel planes in Å with each plane containing $n = 100$ navigation points. Parameter MCKT specifies the initial Boltzmann factor to be used by Monte Carlo algorithm. Parameter MCSTEP specifies the number of steps performed by the Monte Carlo algorithm. Parameter ENDRAD specifies the pore radius value in Å above which the HOLE routine decides that the end of pore has been reached. RASEED parameter resets the random seed for the i-th HOLE routine call.

Section S3. Radial component of the hydropathic imbalance pore function

For scales larger than the lag-domain scale $l_{\alpha=47}(\mathbf{p})$, the magnitude of the HIIS radial field component, $||\vec{\boldsymbol{h}}_{xy}(\mathbf{p}, l_\alpha(\mathbf{p}))||$ (see eq. [m13]), is statistically expected to decrease in relation to its axial counterpart as the median-statistical field descriptor $\langle ||\vec{\boldsymbol{h}}_{xy}(\mathbf{p}, l_\alpha(\mathbf{p}))||/||\vec{\boldsymbol{h}}_z(\mathbf{p}, l_\alpha(\mathbf{p}))||\rangle_\alpha$ becomes smaller than unity and is down-regulated in a fashion that can be roughly described in terms of a power-law function with exponent -0.6628 as shown in Figure S2 (for details regarding the median-statistical calculation see Section S7). This observation suggests that radial hydropathic-field effects exerted upon penetrating ion species

from pore walls of thickness $\langle l_{\alpha=47}(\mathbf{p})\rangle \approx 9.5$ Å cannot be neglected. However, their contribution to the structural stability of the NaV1.7 structural model under scrutiny is expected to be rather marginal for scales larger than $l_{\alpha=47}(\mathbf{p})$ as macroscopically, i.e., for $l_\alpha(\mathbf{p}) \rightarrow L(\mathbf{p})$, $\langle ||\vec{\boldsymbol{h}}_{xy}(\mathbf{p}, l_\alpha(\mathbf{p}))||/||\vec{\boldsymbol{h}}_z(\mathbf{p}, l_\alpha(\mathbf{p}))||\rangle_\alpha$ drops below 0.16 (see inset figure).

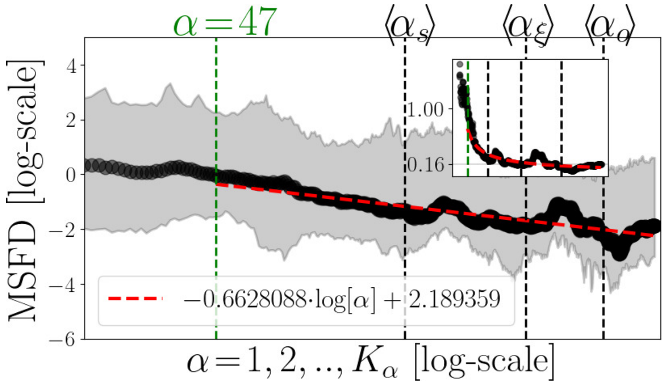

Figure S2: **Scaling behavior of the median-statistical field descriptor $\langle ||\vec{\boldsymbol{h}}_{xy}(\mathbf{p}, l_\alpha(\mathbf{p}))||/||\vec{\boldsymbol{h}}_z(\mathbf{p}, l_\alpha(\mathbf{p}))||\rangle_\alpha$.** **(a)**, A log-vs-log plot of the trace of median-statistical field descriptor (MSFD) $\langle ||\vec{\boldsymbol{h}}_{xy}(\mathbf{p}, l_\alpha(\mathbf{p}))||/||\vec{\boldsymbol{h}}_z(\mathbf{p}, l_\alpha(\mathbf{p}))||\rangle_\alpha$ for $\alpha = 1, 2, .., 800$ is illustrated. $\langle\alpha_s\rangle$, $\langle\alpha_\nu\rangle$, and $\langle\alpha_o\rangle$ designate boundaries among subsequent atom-packing domains around the pore; a lag domain realized for $l_\alpha(\mathbf{p}) \leq s(\mathbf{p})$, an inflection domain consisting of two parts realized for $s(\mathbf{p}) < l_\alpha(\mathbf{p}) \leq \xi(\mathbf{p})$ and $\xi(\mathbf{p}) < l_\alpha(\mathbf{p}) \leq o(\mathbf{p})$, respectively, and an asymptote domain realized for $l_\alpha(\mathbf{p}) > o(\mathbf{p})$ (see Main Text, Methods). Dashed red line corresponds to the best-fitting line on $\log[\langle ||\vec{\boldsymbol{h}}_{xy}(\mathbf{p}, l_\alpha(\mathbf{p}))||/||\vec{\boldsymbol{h}}_z(\mathbf{p}, l_\alpha(\mathbf{p}))||\rangle_\alpha]$-vs-$\log[\alpha]$ data for $\alpha > 47$ with a Pearson coefficient and mean absolute fitting error values of 0.83 and 0.23, respectively. Inset figure illustrates the same data but in linear scale. Shaded area around $\log[\langle ||\vec{\boldsymbol{h}}_{xy}(\mathbf{p}, l_\alpha(\mathbf{p}))||/||\vec{\boldsymbol{h}}_z(\mathbf{p}, l_\alpha(\mathbf{p}))||\rangle_\alpha]$ accounts for 95% confidence intervals.

Section S4. Extracting topological information from cumulative hydropathicity-property function

Topological information from hydropathicity-property functions was extracted according to the following steps [5]:

- Let $f(\mathbf{p}, l_\alpha(\mathbf{p}))$ be a hydropathicity-property function, then if for a given scaling index α there is a pair $\{\mathbf{p}' = \mathbf{p} - \Delta\mathbf{p}, \mathbf{p}\} \in P$ for which the sign-change condition $f(\mathbf{p}', l_\alpha(\mathbf{p}')) \cdot f(\mathbf{p}, l_\alpha(\mathbf{p})) < 0$ is satisfied, extract the four-dimensional point

$$(\mathbf{s} = \mathbf{p}' + \frac{|f(\mathbf{p}', l_\alpha(\mathbf{p}'))|}{|f(\mathbf{p}', l_\alpha(\mathbf{p}'))| + |f(\mathbf{p}, l_\alpha(\mathbf{p}))|} \cdot \Delta\mathbf{p}, l_\alpha(\mathbf{s})) \tag{S2}$$

that represents a zero-crossing point of $f(\mathbf{p}, l_\alpha(\mathbf{p}))$ along $\mathbf{p}$-direction with $|\cdot|$ returning the absolute values of f, and $\mathbf{s}$ approximated via linear interpolation. The set of all detected zero-crossing points of $f(\mathbf{p}, l_\alpha(\mathbf{p}))$ along $\mathbf{p}$-direction for a given scaling index α is represented as $\omega(\alpha)$ so that hydropathic topological information is summarized by the expression

$$\Omega = \bigcup_\alpha \{\omega(\alpha)\} \tag{S3}$$

Section S5. Modeling packing of atoms around NaV1.7's pore

At every $\mathbf{p} \in P$ the GROFIT routine converged to an optimal set of parameter values corresponding to the Richards model and to a special case of it, namely, the Logistic model (Figure S3(a)).

As we show in Figure S3(a), atom-packing conditions around NaV1.7's pore are clearly differentiated among the ES and the IS resulting in a tight packing of atoms at the AG. In particular, density of the atomic environment rapidly increases at the AG as indicated by maximization of the atom-packing rate, $t(\mathbf{p})$, in combination with minimization of the inflection domain's size quantified by $os(\mathbf{p})$ and of the asymptote-value parameter $A(\mathbf{p})$ (Figure S3(a)). In between the CC and the AG, the narrow Logistic model pore region is found capturing the sign-change of $\tilde{q}(\mathbf{p})$ and the transition to the tightly-packed AG environment.

The quality of the modeling approximation is demonstrated by, both, the smallness of the mean absolute fitting error remaining always smaller than 1.3% and of the small uncertainty in parameter values estimation as indicated by narrow confidence intervals around parameter traces (Figure S3(a),(b)).

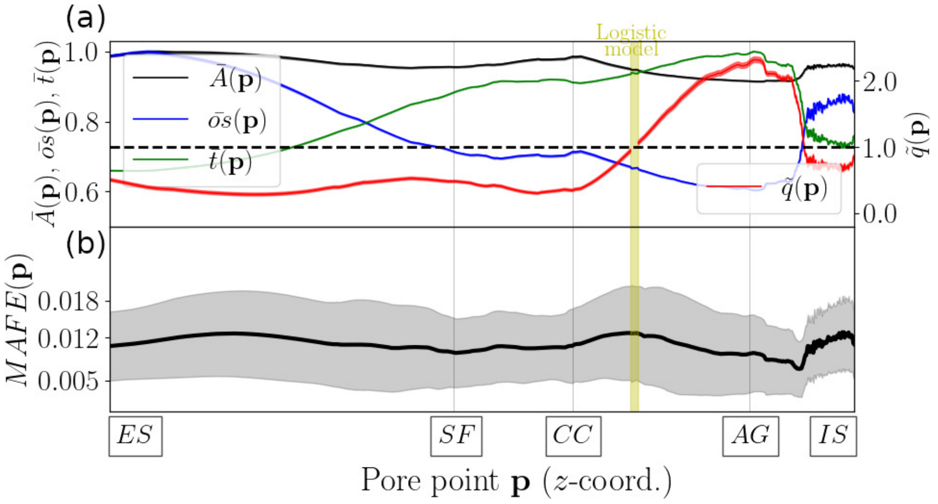

Figure S3: **Model parameters of atom-packing around NaV1.7's pore.** (a), Traces of normalized Richards model parameters $\bar{A}(\mathbf{p})$, $\bar{t}(\mathbf{p})$, $\bar{os}(\mathbf{p})$, and $\tilde{q}(\mathbf{p})$ for $\mathbf{p} \in P$ (see Methods for model parameters definition). Note that for pore points with z-coordinates $\mathrm{p}_z \in [10.5, 11.5]$ the model that best fits atomic CDF data is the Logistic model. (b), Mean absolute fitting error (MAFE) of the Richards model approximation on the normalized CDF trace $\bar{N}(\mathbf{p}, l_\alpha(\mathbf{p}))$ for $\mathbf{p} \in P$. All normalizations were performed with respect to the maximum values of corresponding traces. ES, SF, CC, AG, and IS labels mark the locations of the extracellular side, of the selectivity filter, of the central cavity, of the activation gate, and of the intracellular side, respectively.

Section S6. Modeling the spatial transition from the pore module to the voltage sensors

We consider the NaV1.7 atomic structure to consist of two coupled atomic subsystems, namely, the pore module (PM) and the voltage-sensors (VSs) subsystems. Accordingly, we divided all N_c atoms into two groups, one containing only PM-forming atoms, i.e., atoms forming the S5-S6 helices including their intra/extracellular extensions and the S4-S5 linkers, and one containing only VSs-forming atoms, i.e., atoms forming the S1-S4 helices including their intra/extracellular extensions. The total number of PMs-forming atoms is $N_{PM} = 10109$ and are sampled from the residue sequences P229:K417 (DI), P839:T972 (DII), M1296:G1461 (DIII), and K1617:T1763 (DIV) (note that we consider the S4-S5 linker, as well as, extensions of the PMs to be part of the PM group). On the other hand, the total number of VSs-forming atoms is $N_{VS} = 8458$ and are sampled from the residue sequences L109:I228 (DI), P717:W838 (DII), K1165:G1295 (DIII), and P1485:A1616 (DIV).

The discretized RDF of $N(\mathbf{p}, l_\alpha(\mathbf{p}))$ atoms around $\mathbf{p}$ reads

$$G(\mathbf{p}, l_\alpha(\mathbf{p})) = \frac{\Delta N(\mathbf{p}, l_\alpha(\mathbf{p}))}{\frac{4}{3}\cdot\pi\cdot(l_\alpha(\mathbf{p})^3 - (l'_\alpha(\mathbf{p}))^3)\cdot\rho(\mathbf{p})} \text{ with } l'_\alpha(\mathbf{p}) = l_\alpha(\mathbf{p}) - \Delta l_\alpha(\mathbf{p}) \quad \text{(S4)}$$

where $\Delta l_\alpha(\mathbf{p}) = \frac{L(\mathbf{p}) - \bar{R}(\mathbf{p})}{K_\alpha}$ is the thickness of a spherical shell around $\mathbf{p}$, $\Delta N(\mathbf{p}, l_\alpha(\mathbf{p})) = N(\mathbf{p}, l_\alpha(\mathbf{p})) - N(\mathbf{p}, l'_\alpha(\mathbf{p}))$ is the number of atoms found within the spherical shell of thickness $\Delta l_\alpha(\mathbf{p})$ centered at $\mathbf{p}$ and $\rho(\mathbf{p}) = \frac{N_c}{V(\mathbf{p})} = \frac{N_c}{\frac{4}{3}\cdot\pi\cdot(L(\mathbf{p}) - R(\mathbf{p}))^3}$ is the average atomic density around $\mathbf{p}$.

Using equation S4 we approximated the RDFs of the PM and VS atoms around $\mathbf{p}$ with

$$G_{PM}(\mathbf{p}, l_\alpha(\mathbf{p})) = \frac{\Delta n_{PM}(\mathbf{p}, l_\alpha(\mathbf{p}))}{\frac{4}{3}\cdot\pi\cdot(l_\alpha(\mathbf{p})^3 - (l'_\alpha(\mathbf{p}))^3)\cdot\rho_{PM}(\mathbf{p})} \quad \text{(S5)}$$

and

$$G_{VS}(\mathbf{p}, l_\alpha(\mathbf{p})) = \frac{\Delta n_{VS}(\mathbf{p}, l_\alpha(\mathbf{p}))}{\frac{4}{3}\cdot\pi\cdot(l_\alpha(\mathbf{p})^3 - (l'_\alpha(\mathbf{p}))^3)\cdot\rho_{VS}(\mathbf{p})} \quad \text{(S6)}$$

, respectively, where $N(\mathbf{p}, l_\alpha(\mathbf{p})) = N_{PM}(\mathbf{p}, l_\alpha(\mathbf{p})) + N_{VS}(\mathbf{p}, l_\alpha(\mathbf{p}))$, $\rho_{PM}(\mathbf{p}) = \frac{N_{PM}}{V(\mathbf{p})}$ and $\rho_{VS}(\mathbf{p}) = \frac{N_{VS}}{V(\mathbf{p})}$.

In order to obtain a relative measure of how the RDF of PM atoms varies with respect to the RDF of VS atoms, and vice versa, we used the PMs-VSs equilibrium RDF [5, 6]

$$e(\mathbf{p}, l_\alpha(\mathbf{p})) = sm(G_{PM}(\mathbf{p}, l_\alpha(\mathbf{p})) - G_{VS}(\mathbf{p}, l_\alpha(\mathbf{p}))) \tag{S7}$$

where $sm(\cdot)$ implements the Nadaraya-Watson kernel regression R-function [7] with a bandwidth parameter value of $bw = 15$. $e(\mathbf{p}, l_\alpha(\mathbf{p})$ is interpreted as the smoothed probability of finding a PM atom instead of a VS atom, and vice-versa, at distance $l_\alpha(\mathbf{p})$ from $\mathbf{p}$. Specifically, if $e(\mathbf{p}, l_\alpha(\mathbf{p})) > 0$ ($e(\mathbf{p}, l_\alpha(\mathbf{p})) < 0$) then the smoothed probability of finding a PM atom within the spherical shell of width $\Delta l_\alpha(\mathbf{p})$ is larger than that of finding a VS (PM) atom. Accordingly, what is of interest here is the sign-change behavior of $e(\mathbf{p}, l_\alpha(\mathbf{p})$ for increasing $l_\alpha(\mathbf{p})$. We investigated it by detecting for every $\mathbf{p}$ the pair $\{l'_\alpha(\mathbf{p}), l_\alpha(\mathbf{p})\}$ for which the sign-change condition $e(\mathbf{p}, l'_\alpha(\mathbf{p})) \cdot e(\mathbf{p}, l_\alpha(\mathbf{p})) < 0$ is satisfied and approximating with linear interpolation the root location

$$\nu(\mathbf{p}) = l'_\alpha(\mathbf{p}) - \frac{e(\mathbf{p}, l'_\alpha(\mathbf{p}))}{e(\mathbf{p}, l_\alpha(\mathbf{p})) - e(\mathbf{p}, l'_\alpha(\mathbf{p}))} \cdot \Delta l_\alpha(\mathbf{p}) \tag{S8}$$

where $e(\mathbf{p}, l_\alpha(\mathbf{p}))$ changes sign along $l_\alpha(\mathbf{p})$-direction.

Section S7. Statistical representation of scalar functions

Throughout this study $\langle \cdot \rangle$ represents a statistical operator returning the median of the data set upon which it operates. Note that the choice of the median as a statistical measure reflects the fact that no assumption has been made for the distribution of data set values.

Statistical representation of scalar function $f(\mathbf{p}, l_\alpha(\mathbf{p}))$. A statistical representation of the scalar function $f(\mathbf{p}, l_\alpha(\mathbf{p}))$ for a given α was obtained in terms of $\langle f(\mathbf{p}, l_\alpha(\mathbf{p}))\rangle_\alpha$ where the subscript α indicates that the statistical operator acts for a given α, i.e., on the set of values $F(\alpha) = \{f(\mathbf{p}, l_\alpha(\mathbf{p})) \mid \mathbf{p} \in P\}$.

Statistical representations of scalar functions $s(\mathbf{p})$, $\nu(\mathbf{p})$, $\xi(\mathbf{p})$, and $o(\mathbf{p})$. Statistical representations of the functions $s(\mathbf{p})$, $\nu(\mathbf{p})$, $\xi(\mathbf{p})$, and $o(\mathbf{p})$ were obtained in two steps;

- *Step 1.* Let $f(\mathbf{p})$ represent one of the aforementioned scalar functions, then, for every $\mathbf{p}$ find the α indices for which $|f(\mathbf{p}) - l_\alpha(\mathbf{p})|$ is minimized, i.e., $\alpha_f = \{\alpha \mid \underset{\alpha \in A}{min}(|f(\mathbf{p}) - l_\alpha(\mathbf{p})|)\}$.

- *Step 2.* Calculate the median $\langle \alpha_f \rangle$ of the data set α_f. Note that the minimum and the maximum value contained in α_f is given by $min(\alpha_f)$ and $max(\alpha_f)$, respectively.

Section S8. Missense *SCN9A*-gene mutations

A collection of well-studied gain-of-function (GOF) *SCN9A*-gene mutations are introduced which have been experimentally verified to be causally related with IEM, SFN, and PEPD phenotypes (Table S2). A collection of neutrals *SCN9A*-gene mutations is incorporated from [8] containing variants not causing biophysical abnormalities (nBABNs) and homologous single-nucleotide polymorphisms (hSNPs) (Table S3). In addition, we consider as neutrals four variants which have been previously classified as non-pathogenic based on procedures described in [9] (see caption of Table S3). Selection criterion for hSNPs is that they share at least 90% nucleotide sequence identity with *SCN9A* homologous genes (see also [10, 11, 12]). The search for hSNPs was implemented in the NCBI HomoloGene Database [13]. All nBABNs cases are associated with *in vitro* observations (see caption of Table S3).

What is of interest for this study is the location of the mutated residue (i.e., mutation site) within the NaV1.7 structure which is calculated by

$$\mathbf{v} = \frac{1}{M_{res.}} \sum_{i}^{n_{res.}} m_i^{res.} \cdot \mathbf{c}_i^{res.} \tag{S9}$$

where $\mathbf{c}_i^{res.}$ is the center and $m_i^{res.}$ is the mass of the i-th atom belonging to the mutated residue, $M_{res.} = \sum_i^{n_{res.}} m_i^{res.}$ is the total residue mass, and $n_{res.}$ is the total number of atoms forming the residue.

The mutation structural location is mapped on two dimensions by rounding its z-coordinate, v_z, to SAMPLE accuracy (see Table S1) so that it can be assigned to a pore point z-coordinate. Then, the distance between the rounded mutation structural location and its assigned pore point determines the location of the mutation site on contour maps of Figures 2, 3 and 5(a) appearing in the Main Text.

Mutation	Reference	Mutation	Reference
I136V (IEM)	[14, 15]	A1632T (IEM)	[43]
S211P (IEM)	[16]	A1632G (IEM)	[44]
F216S (IEM)	[17]	A1746G (IEM)	[42]
I234T (IEM)	[18]		
S241T (IEM)	[19, 20]	V1298F (PEPD)	[45, 38]
L245V (IEM)	[21]	V1299F (PEPD)	[46]
N395K (IEM)	[22]	I1461T (PEPD)	[47]
V400M (IEM)	[23]	G1607R (PEPD)	[48]
L823R (IEM)	[24]	L1612P (PEPD)	[49]
F826Y (IEM)	[25]	M1627K (PEPD)	[50, 47, 46]
I848T (IEM)	[26, 27, 28, 15]	A1632E (PEPD)	[51]
G856R (IEM)	[29]		
G856D (IEM)	[30]	R185H (SFN)	[52]
L858H (IEM)	[31, 32, 28]	I228M (SFN)	[53]
L858F (IEM)	[33]	I720K (SFN)	[54]
A863P (IEM)	[34]	I739V (SFN)	[52]
V872G (IEM)	[35]	M932L (SFN)	[54]

Q875E (IEM)	[36]	R1279P (SFN)	[55]
L955Del (IEM)	[37]	T1596I (SFN)	[56]
P1308L (IEM)	[38]		
V1316A (IEM)	[39, 40]		
F1449V (IEM)	[41]		
W1538R (IEM)	[42]		

Table S2: **Pain-related missense *SCN9A*-gene mutations.** Missense *SCN9A*-gene mutations related with IEM, PEPD, and SFN pain disease. References hint to the published study describing biophysical attributes of the corresponding mutation.

Mutation	Type	Mutation	Type
S126A	hSNP	L127A	hSNP
M145L	hSNP	N146S	hSNP
V194I	hSNP	L201V	hSNP
N206D	hSNP	E759D	hSNP
A766T	hSNP	A766V	hSNP
I767V	hSNP	V795I	hSNP
A815S	hSNP	K1176R	hSNP
R1207K	hSNP	T1210N	hSNP
I1235V	hSNP	A1505V	hSNP
S1509T	hSNP	S1509A	hSNP
Q1530P	hSNP	Q1530K	hSNP
Q1530D	hSNP	H1531Y	hSNP
M1532V	hSNP	E1534D	hSNP
Y1537N	hSNP	T1548S	hSNP
H1560Y	hSNP	H1560C	hSNP
V1565I	hSNP	I1577L	hSNP
D1586E	hSNP	T1590K	hSNP

T1590R	hSNP	V1613I	hSNP
D890E	hSNP	D890V	hSNP
T1398M	hSNP	I1399D	hSNP
D1411S	hSNP	D1411N	hSNP
K1412I	hSNP	K1415I	hSNP
D1662A	hSNP		
K1700A	hSNP	N1245S [57, 58]	nBABN
D1674A	hSNP	L1267V [57]	nBABN
S1419N	hSNP	V1428I	nBABN
K1412E	hSNP	T920N	nBABN

Table S3: **Neutral missense *SCN9A*-gene mutations.** Neutral missense *SCN9A*-gene mutations are not expected to associate with pain disease phenotypes, and consist of two subgroups; a hSNPs group and a nNABNs group incorporated from [8]. V1428I and T920N nNABNs represent unpublished experimental observations of the PROPANE consortium. Additionally, four more neutrals are introduced, namely, M130I, M787V, S802G and V810M, which were previously characterized by PROPANE consortium as non-pathogenic in accordance to classification procedures described in [9].

Section S9. Distance metrics for classification of mutation sites

S9a. Estimating distance between mutation structural locations and HP's boundary

The distance between a mutation structural location, $\mathbf{v}$, and the HP's boundary, $\Omega^{(0)}$, is estimated by

$$D_{HP}(\mathbf{v}) = \langle\{|\ ||\mathbf{v} - \mathbf{s}^{(0)}|| - l_\alpha(\mathbf{s}^{(0)})\ |\ |\ (\mathbf{s}^{(0)}, l_\alpha(\mathbf{s}^{(0)})) \in \Omega^{(0)}\}\rangle \qquad \text{(S10)}$$

representing the statistical-median value of the shortest euclidean distances between $\mathbf{v}$ and the surfaces of the spheres of radius $l_\alpha(\mathbf{s}^{(0)})$ centered at $\mathbf{s}^{(0)}$ (see eq. S2).

S9b. Estimating distance between mutation structural locations and inflection points

The distance between a mutation structural location, $\mathbf{v}$, and an inflection point, $\xi(\mathbf{p})$, is estimated by

$$D_{\xi(\mathbf{p})}(\mathbf{v}) = |\ ||\mathbf{v} - \mathbf{p}|| - \xi(\mathbf{p})\ | \tag{S11}$$

corresponding to the shortest euclidean distance of $\mathbf{v}$ from the surface of the inflection sphere of radius $\xi(\mathbf{p})$ centered at $\mathbf{p}$.

For a collection of mutation structural locations (e.g., for the collection of GOF mutation structural locations), let it be V, equation S11 is computed for every $\mathbf{v} \in V$, and the statistical-median value $D_{\xi(\mathbf{p})}(V) = \langle\{|\ ||\mathbf{v} - \mathbf{p}|| - \xi(\mathbf{p})\ |\ |\ \mathbf{v} \in V\}\rangle$ is employed in order to statistically describe the distance of mutation structural locations from $\xi(\mathbf{p})$.

Section S10. In the search of critical pore regions; scaling analysis of HIIS axial field component

In order to detect critical signatures of HIs around NaV1.7's pore, we quantified linearity of $\log[||\vec{\boldsymbol{m}}_z^{(1)}(\mathbf{p}, l_\alpha(\mathbf{p}))||]$-vs-$\log[l_\alpha(\mathbf{p})]$ within the lag domain interval, i.e., for $D(\mathbf{p}) < l_\alpha(\mathbf{p}) \leq s(\mathbf{p})$, and within the first- and second-part of the inflection domain, i.e., for $s(\mathbf{p}) < l_\alpha(\mathbf{p}) \leq \xi(\mathbf{p})$ and $\xi(\mathbf{p}) < l_\alpha(\mathbf{p}) \leq o(\mathbf{p})$, respectively, by fitting the line $\gamma(\mathbf{p}) \cdot \log[l_\alpha(\mathbf{p})] + \beta(\mathbf{p})$ where the goodness of the fitting for each domain interval was estimated in terms of the corresponding Pearson coefficient score, $PC(\mathbf{p})$, and corresponding fitting error. Noteworthy, for cases where limited data are available (such as the case presented in this study) usage of fitting techniques upon well-defined intervals is advised in order to eliminate numerical biases [59]. To do that, we fetched the least square fitting algorithm implemented within the R [7] environment and proceeded with extracting power-law exponents from log-vs-log plots. Linear fittings on asymptote domain intervals (i.e., for $o(\mathbf{p}) < l_\alpha(\mathbf{p}) \leq L(\mathbf{p})$) were not considered as asymptote domain does not contain any mutations sites (see Main Text)

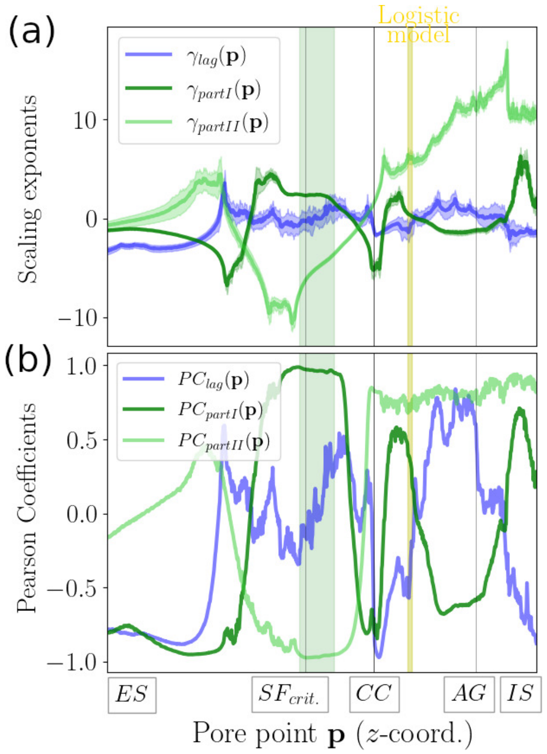

Figure S4: **Scaling analysis of HIIS axial field component.** (a), Traces of power-law exponents $\gamma_{lag}(\mathbf{p})$, $\gamma_{partI}(\mathbf{p})$ and $\gamma_{partII}(\mathbf{p})$ describing the scaling behavior of the HIIS axial field component, (see Methods, eq. [m13]), within the lag domain (i.e., for $l_\alpha(\mathbf{p}) \leq s(\mathbf{p})$), the first part of the inflection domain (i.e., for $s(\mathbf{p}) < l_\alpha(\mathbf{p}) \leq \xi(\mathbf{p})$), and the second part of the inflection domain (i.e., for $\xi(\mathbf{p}) < l_\alpha(\mathbf{p}) \leq o(\mathbf{p})$) respectively, are plotted for $\mathbf{p} \in P$. Shaded area around the curves indicates uncertainty in exponent quantification based on fitting error analysis. (b), Traces of corresponding Pearson coefficients $PC_{lag}(\mathbf{p})$, $PC_{partI}(\mathbf{p})$ and $PC_{partII}(\mathbf{p})$ are plotted for $\mathbf{p} \in P$. Note that $0 \leq |PC(\mathbf{p})| \leq 1$ always holds with a value close to 1.0 indicating a "good" power-law approximation. Green-shaded area around $SF_{crit.}$ pore point indicates the pore region $-13.4 \leq \mathrm{p}_z \leq -5.8$ where the product $|PC_{partI}(\mathbf{p})| \cdot |PC_{partII}(\mathbf{p})|$ attains its largest values as, both, $|PC_{partI}(\mathbf{p})|$ and $|PC_{partII}(\mathbf{p})|$ attain values higher than 0.95. This is indicative of maximization of the "goodness" of the power-law approximation. ES, SF_{crit}, CC, AG, and IS labels mark the locations of the extracellular side, of the critical

pore point $\mathbf{p}_{crit.}$, of the central cavity, of the activation gate, and of the intracellular side, respectively.

Traces of Pearson coefficients are shown in Figure S4 where we show that, both, $|PC_{partI}(\mathbf{p})|$ and $|PC_{partII}(\mathbf{p})|$ attain values higher than 0.95 within the SF interval $-13.4 \le \mathrm{p}_z \le -5.8$ where corresponding scaling exponents $\gamma(\mathbf{p})_{partI}$ and $\gamma(\mathbf{p})_{partII}$ reveal an up- and down-regulation of HIIS axial field component before and after the inflection point, respectively. Accordingly, the power-law scheme described by equation [m14] (see Main Text, Methods) is accurately reproduced for pore points $-13.4 \le \mathrm{p}_z \le -5.8$ where maximization of the product $|PC_{partI}(\mathbf{p})| \cdot |PC_{partII}(\mathbf{p})|$ occurs at $\mathbf{p}_{crit.} = (\mathrm{p}_x \approx -2.58, \mathrm{p}_y \approx -0.06, \mathrm{p}_z = -12.1)$ (see Figure S4, and Main Text, Figure 5(a)). Note that the trace of $\gamma_{lag}(\mathbf{p})$ remains close to zero while the magnitude of its Pearson coefficient attains values higher than 0.95 for a short interval in the vicinity of the CC thus not allowing for a robust power-law approximation of HIIS axial field component within the lag domain.

Section S11. Classification of *SCN9A*-gene mutations based on a weighted topological-distance average measure

We calculated the weighted topological-distance average $w_{HP} \cdot D_{HP}(\mathbf{v}) + w_\xi \cdot D_{\xi(\mathbf{p}_{crit.})}(\mathbf{v})$, where w_{HP} and w_ξ are weights, for all the pain-related and neutral mutation structural locations, and fed retrieved distances into a binary classifier. The quality of retrieved classifications is demonstrated in Figure S5 in terms of the area under the ROC curve for $w_\xi = 1 - w_{HP} = i \cdot 0.001$, $i = 0, 1, .., 1000$. The linear combination of $D_{HP}(\mathbf{v})$ with $D_{\xi(\mathbf{p}_{crit.})}(\mathbf{v})$ which maximized the area under the ROC curve corresponds to $w_\xi = 0.618$ and $w_{HP} = 0.382$ (Figure S5).

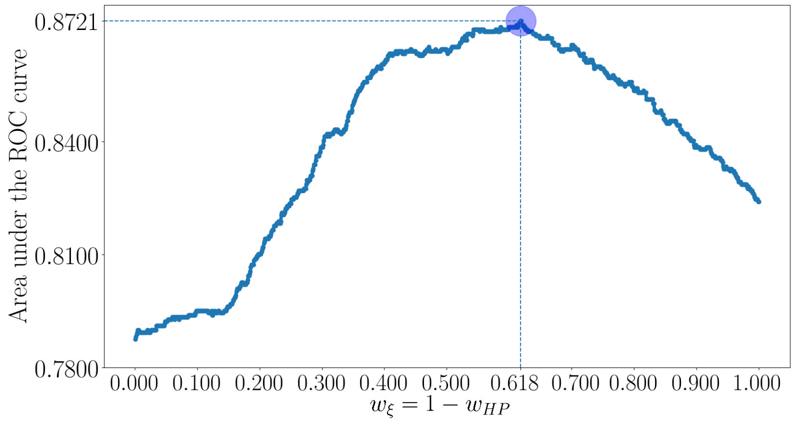

Figure S5: **Area under ROC curve retrieved from linear combination of topological distances $D_{HP}(\mathbf{v})$ and $D_{\xi(\mathbf{p}_{crit.})}(\mathbf{v})$.** The area under the ROC curve is plotted for different weights $w_\xi = 1 - w_{HP} = i \cdot 0.001$, $i = 0, 1, .., 1000$ determining the weighted topological-distance average $w_{HP} \cdot D_{HP}(\mathbf{v}) + w_\xi \cdot D_{\xi(\mathbf{p}_{crit.})}(\mathbf{v})$ where $D_{HP}(\mathbf{v})$ and $D_{\xi(\mathbf{p}_{crit.})}(\mathbf{v})$ are given by equations S10 and S11, respectively.

Declarations

Ethics approval and consent to participate

Not applicable.

Consent for publication

Not applicable.

Availability of data and materials

Data sharing is not applicable to this article as no datasets were generated or analyzed during the current study. The 3D structural model of the NaV1.7 channel is available from the authors with permission of YY and SGW.

Competing interests

The authors declare that they have no competing interests.

Funding

The study was partly funded by the European Union 7th Framework Programme (grant n602273).

Authors' contributions

MNX designed the study, performed computations, and analyzed the data; DK, RW and PL contributed to refinement of algorithmic procedures; YY provided with the 3D structural model of the NaV1.7 channel; MMG contributed to variants selection and classification; RW, PL, DK, YY, JH, HJS and SGW provided with critical feedback and helped with the interpretation of the results; YY and SGW encouraged MNX to focus on specific aspects of the findings; HJS supervised the study; GL and CGF were in charge of overall direction; MNX wrote the manuscript in consultation with all the co-authors; All co-authors have critically revised the manuscript.

Acknowledgments

We acknowledge technical and scientific support provided by the PROPANE study group.

References

[1] Shen, H., Liu, D., Wu, K., Lei, J., Yan, N. Structures of human $Na_V1.7$ channel in complex with auxiliary subunits and animal toxins. *Science* **363**, 1303-1308 (2019).

[2] Konagurthu, A.S., Whisstock, J.C., Stuckey, P.J., Lesk, A.M. MUSTANG: A multiple structural alignment algorithm. *Proteins* **64**, 559-574 (2006).

[3] Needleman, S.B. Wunsch, C.D. A general method applicable to the search for similarities in the amino acid sequence of two proteins. *J Mol Biol* **48**, 443-53 (1970).

[4] Kapcha, L.H., Rossky, P.J. A simple atomic-level hydrophobicity scale reveals protein interfacial structure. *J Mol Biol* **426**, 484-498 (2014).

[5] Xenakis, M.N., Kapetis, D., Yang, Y. et al. Cumulative hydropathic topology of a voltage-gated sodium channel at atomic resolution. *Proteins* **88**, 1319-1328 (2020).

[6] Xenakis, M.N., Kapetis, D., Yang, Y. et al. Non-extensitivity and criticality of atomic hydropathicity around a voltage-gated sodium channel's pore; a modeling study. *J Biol Phys* **47**, 61-77 (2021).

[7] R Core Team *R: A language and environment for statistical computing.* R Foundation for Statistical Computing, Vienna, Austria. http://www.R-project.org/ (2014).

[8] Kapetis, D., Yang, Y., Sassone, J. et al. Network topology of NaV1.7 mutations in sodium channel-related painful disorders. *BMC Systems Biology* **11**, 28 (2017).

[9] Wallis, Y., Payne, S., McAnulty, C. et al. Practice guidelines for the evaluation of pathogenecity and reporting of sequence variants in clinical molecular genetics. *ACGS* (2013). http://www.acgs.uk.com/media/774853/evaluation_and_reporting_of_sequence_variants_bpgs_june_2013_-_finalpdf.pdf (accessed May 25, 2013).

[10] Care, M.A., Needham, C.J., Bulpitt, A.J., Westhead D.R. Deleterious SNP prediction:Be mindful of your training data! *Bioinformatics* **23**, 664-72 (2007).

[11] Sunyaev, S., Ramensky, V., Koch, I., Lathe, W., Kondrashov, A.S., Bork, P. Prediction of deleterious human alleles. *Hum Mol Genet* **10**, 591-7 (2001).

[12] Yue, P., Moult, J. Identification and analysis of deleterious human SNPs. *J Mol Biol* **356**, 1263-74 (2006).

[13] NCBI Resource Coordinators. Database resources of the National Center for Biotechnology Information. *Nucleic Acids Res* Jan 4;44(D1):D7-19 (2016). doi: 10.1093/nar/gkv1290.Epub2015Nov28.PMID:26615191; PMCID:PMC4702911

[14] Cheng, X., Dib-Hajj, S.D., Tyrrell, L., Waxman, S.G. Mutation I136V alters electrophysiological properties of the Na(v)1.7 channel in a family with onset of erythromelalgia in the second decade. *Mol Pain* **4**, 1 (2008).

[15] Estacion, M., Choi, J.S., Eastman, E.M. et al. Can robots patch-clamp as well as humans? Characterization of a novel sodium channel mutation. *J Physiol* **588**, 1915-1927 (2010).

[16] Wu, M.T., Huang, P.Y., Yen, C.T., Chen, C.C., Lee, M.J. A novel SCN9A mutation responsible for primary erythromelalgia and is resistant to the treatment of sodium channel blockers. *PLoS One* **8**, e55212 (2013).

[17] Choi, J.S., Dib-Hajj, S.D., Waxman, S.G. Inherited erythermalgia: limb pain from an S4 charge-neutral Na channelopathy. *Neurology* **67**, 1563-1567 (2006).

[18] Ahn, H.S., Dib-Hajj, S.D., Cox, J.J. et al. A new Nav1.7 sodium channel mutation I234T in a child with severe pain. *Eur J Pain* **14**, 944-950 (2010).

[19] Lampert, A., Dib-Hajj, S.D., Tyrrell, L., Waxman, S.G. Size matters: Erythromelalgia mutation S241T in Nav1.7 alters channel gating. *J Biol Chem* **281**, 36029-36035 (2006).

[20] Yang,,Y., Dib-Hajj, S.D., Zhang, J. et al. Structural modelling and mutant cycle analysis predict pharmacoresponsiveness of a Na(V)1.7 mutant channel. *Nat Commun* **3**, 1186 (2012).

[21] Emery, E.C., Habib, A.M., Cox, J.J. et al. Novel SCN9A mutations underlying extreme pain phenotypes: unexpected electrophysiological and clinical phenotype correlations. *J Neurosci* **35**, 7674-7681 (2015).

[22] Sheets, P.L., Jackson, J.O. 2nd, Waxman, S.G., Dib-Hajj, S.D., Cummins, T.R. A Nav1.7 channel mutation associated with hereditary erythromelalgia contributes to neuronal hyperexcitability and displays reduced lidocaine sensitivity. *J Physiol* **581**, 1019-1031 (2007).

[23] Fischer, T.Z., Gilmore, E.S., Estacion, M. et al. A novel Nav1.7 mutation producing carbamazepine-responsive erythromelalgia. *Ann Neurol* **65**, 733-741 (2009).

[24] Lampert, A., Dib-Hajj, S.D., Eastman, E.M., Tyrrell, L., Lin, Z., Yang, Y., Waxman, S.G. Erythromelalgia mutation L823R shifts activation and inactivation of threshold sodium channel Nav1.7 to hyperpolarized potentials. *Biochem Biophys Res Commun* **390**, 319-324 (2006).

[25] Wu, B., Zhang, Y., Tang, H. et al. A Novel SCN9A Mutation (F826Y) in Primary Erythromelalgia Alters the Excitability of Nav1.7. *Curr Mol Med* **17**, 450-457 (2017).

[26] Cummins, T.R., Dib-Hajj, S.D., Waxman, S.G. Electrophysiological properties of mutant Nav1.7 sodium channels in a painful inherited neuropathy. *J Neurosci* **24**, 8232-8236 (2004).

[27] Han, C., Dib-Hajj, S.D., Lin, Z. et al. Early- and late-onset inherited erythromelalgia: genotype-phenotype correlation. *Brain* **132**, 1711-1722 (2009).

[28] Theile, J.W., Cummins, T.R. Inhibition of Navβ4 peptide-mediated resurgent sodium currents in Nav1.7 channels by carbamazepine, riluzole, and anandamide. *Mol Pharmacol* **80**, 724-734 (2011).

[29] Tanaka, B.S., Nguyen, P.T., Zhou, E.Y. et al. Gain-of-function mutation of a voltage-gated sodium channel NaV1.7 associated with peripheral pain and impaired limb development. *J Biol Chem* **292**, 9262-9272 (2017).

[30] Hoeijmakers, J.G., Han, C., Merkies, I.S. et al. Small nerve fibres, small hands and small feet: a new syndrome of pain, dysautonomia and acromesomelia in a kindred with a novel NaV1.7 mutation. *Brain* **135**, 345-358 (2012).

[31] Cummins, T.R., Dib-Hajj, S.D., Waxman, S.G. Electrophysiological properties of mutant Nav1.7 sodium channels in a painful inherited neuropathy. *J. Neurosci* **24**, 8232-8236 (2004).

[32] Rush, A.M., Dib-Hajj, S.D., Liu, S., Cummins, T.R., Black, J.A., Waxman, S.G. A single sodium channel mutation produces hyper- or hypoexcitability in different types of neurons. *Proc Nat Acad Sci USA* **103**, 8245-8250 (2006).

[33] Han, C., Rush, A.M., Dib-Hajj, S.D. et al. Sporadic onset of erythermalgia: a gain-of-function mutation in Nav1.7. *Ann Neurol* **59**, 553-558 (2006).

[34] Harty, T.P., Dib-Hajj, S.D., Tyrrell, L., Blackman, R., Hisama, F.M., Rose, J.B., Waxman, S.G. Na_V1.7 mutant A863P in erythromelalgia: effects of altered activation and steady-state inactivation on excitability of nociceptive dorsal root ganglion neurons. *J Neurosci* **26**, 12566-12575 (2006).

[35] Choi, J.S., Zhang, L., Dib-Hajj, S.D. et al. Mexiletine-responsive erythromelalgia due to a new Na(v)1.7 mutation showing use-dependent current fall-off. *Exp Neurol* **216**, 383-389 (2009).

[36] Stadler, T., O'Reilly, A.O., Lampert, A. Erythromelalgia mutation Q875E Stabilizes the activated state of sodium channel Nav1.7. *J Biol Chem* **290**, 6316-6325 (2015).

[37] Cheng, X., Dib-Hajj, S.D., Tyrrell, L., Te Morsche, R.H., Drenth, J.P., Waxman, S.G. Deletion mutation of sodium channel Na(V)1.7 in inherited erythromelalgia: enhanced slow inactivation modulates dorsal root ganglion neuron hyperexcitability. *Brain* **134**, 1972-1986 (2011).

[38] Cheng, X., Dib-Hajj, S.D., Tyrrell, L., Wright, D.A., Fischer, T.Z., Waxman, S.G. Mutations at opposite ends of the DIII/S4-S5 linker of sodium channel NaV1.7 produce distinct pain disorders. *Mol Pain* **6**, 24 (2010).

[39] Estacion, M., Yang, Y., Dib-Hajj, S.D. et al. A new Nav1.7 mutation in an erythromelalgia patient. *Biochem Biophys Res Commun* **432**, 99-104 (2013).

[40] Wu, M.T., Huang, P.Y., Yen, C.T., Chen, C.C., Lee, M.J. A novel SCN9A mutation responsible for primary erythromelalgia and is resistant to the treatment of sodium channel blockers. *PLoS One* **8**, e55212 (2013).

[41] Dib-Hajj, S.D., Rush, A.M., Cummins, T.R. et al. Gain-of-function mutation in Nav1.7 in familial erythromelalgia induces bursting of sensory neurons. *Brain* **128**, 1847-1854 (2005).

[42] Cregg, R., Laguda, B., Werdehausen, R. et al. Novel mutations mapping to the fourth sodium channel domain of Nav1.7 result in variable clinical manifestations of primary erythromelalgia. *Neuromolecular Med* **15**, 265-278 (2013).

[43] Eberhardt, M., Nakajima, J., Klinger, A.B. et al. Inherited pain: sodium channel Nav1.7 A1632T mutation causes erythromelalgia due to a shift of fast inactivation. *J Biol Chem* **289**, 1971-1980 (2014).

[44] Yang, Y., Huang, J., Mis, M.A. et al. Nav1.7-A1632G Mutation from a Family with Inherited Erythromelalgia: Enhanced Firing of Dorsal Root Ganglia Neurons Evoked by Thermal Stimuli. *J Neurosci* **36**, 7511-7522 (2016).

[45] Jarecki, B.W., Sheets, P.L., Jackson, J.O. 2nd, Cummins, T.R. Paroxysmal extreme pain disorder mutations within the D3/S4-S5 linker of Nav1.7 cause moderate destabilization of fast inactivation. *J Physiol* **586**, 4137-4153 (2008).

[46] Theile, J.W, Jarecki, B.W., Piekarz, A.D., Cummins, T.R. Nav1.7 mutations associated with paroxysmal extreme pain disorder, but not erythromelalgia, enhance Navβ4 peptide-mediated resurgent sodium currents. *J Physiol* **589**, 597-608 (2011).

[47] Fertleman, C.R., Baker, M.D., Parker, K.A. et al. SCN9A mutations in paroxysmal extreme pain disorder: allelic variants underlie distinct channel defects and phenotypes. *Neuron* **52**, 767-774 (2006).

[48] Choi, J.S., Boralevi, F., Brissaud, O. et al. Paroxysmal extreme pain disorder: a molecular lesion of peripheral neurons. *Nat Rev Neurol* **7**, 51-55 (2011).

[49] Suter, M.R., Bhuiyan, Z.A., Laedermann, C.J. et al. p.L1612P, a novel voltage-gated sodium channel Nav1.7 mutation inducing a cold sensitive paroxysmal extreme pain disorder. *Anesthesiology* **122**, 414-423 (2015).

[50] Dib-Hajj, S.D., Estacion, M., Jarecki, B.W. et al. Paroxysmal extreme pain disorder M1627K mutation in human Nav1.7 renders DRG neurons hyperexcitable. *Mol Pain* **4**, 37 (2008).

[51] Estacion, M., Dib-Hajj, S.D., Benke, P.J. et al. NaV1.7 gain-of-function mutations as a continuum: A1632E displays physiological changes associated with erythromelalgia and paroxysmal extreme pain disorder mutations and produces symptoms of both disorders. *J Neurosci* **28**, 1079-11088 (2008).

[52] Han, C., Hoeijmakers, J.G., Liu, S. et al. Functional profiles of SCN9A variants in dorsal root ganglion neurons and superior cervical ganglion neurons correlate with autonomic symptoms in small fibre neuropathy. *Brain* **135**, 2613-2628 (2012).

[53] Estacion, M., Han, C., Choi, J.S. et al. Intra- and interfamily phenotypic diversity in pain syndromes associated with a gain-of-function variant of NaV1.7. *Mol Pain* **7**, 92 (2011).

[54] Faber, C.G., Hoeijmakers, J.G., Ahn, H.S. et al. Gain of function Nav1.7 mutations in idiopathic small fiber neuropathy. *Ann. Neurol.* **71**, 26-39 (2012).

[55] Huang, J., Yang, Y., Dib-Hajj, S.D. et al. Depolarized inactivation overcomes impaired activation to produce DRG neuron hyperexcitability in a Nav1.7 mutation in a patient with distal limb pain. *J Neurosci* **34**, 12328-12340 (2014).

[56] Blesneac, I., Themistocleous, A.C., Fratter, C. et al. Rare NaV1.7 variants associated with painful diabetic peripheral neuropathy. *Pain* **159**, 469-480 (2018).

[57] Brouwer, B.A., Merkies, I.S.J., Gerrits, M.M., Waxman, S.G, Hoeijmakers J.G.J., Faber, C.G. Painful neuropathies: the emerging role of sodium channelopathies. *J Peripher Nerv Syst* **19**, 53-65 (2014).

[58] Le, Cann, K., Meents, J.E., Eswaran, V.S.B. Assessing the impact of pain-linked Nav1.7 variants: An example of two variants with no biophysical effect. *Channels* **15**, 208-228 (2021).

[59] Moretti, P., Muñoz, M.A. Griffiths phases and the stretching of criticality in brain networks. *Nat Commun* **4**, 2521 (2013).
